# TAK1 mediates neuronal pyroptosis in early brain injury after subarachnoid hemorrhage

**DOI:** 10.1186/s12974-021-02226-8

**Published:** 2021-08-30

**Authors:** Pengfei Xu, Chunrong Tao, Yuyou Zhu, Guoping Wang, Lingqi Kong, Wenyu Li, Rui Li, Juanji Li, Chao Zhang, Li Wang, Xinfeng Liu, Wen Sun, Wei Hu

**Affiliations:** 1 Stroke Center & Department of Neurology, The First Affiliated Hospital of USTC, Division of Life Sciences and Medicine, University of Science and Technology of China, Hefei, 230036 Anhui China; 2 The First Affiliated Hospital of USTC, Division of Life Sciences and Medicine, University of Science and Technology of China, Hefei, 230036 Anhui China; 3 Department of Neurology, Jinling Hospital, Medical School of Nanjing University, Nanjing, 210002 Jiangsu China

**Keywords:** Subarachnoid hemorrhage, TAK1, Pyroptosis, NLRP3 inflammasome, ROS

## Abstract

**Background:**

Innate immunity can facilitate early brain injury (EBI) following subarachnoid hemorrhage (SAH). Numerous studies suggest that pyroptosis could exacerbate extracellular immune responses by promoting secretion of inflammatory cytokines. Transforming growth factor-β-activated kinase 1 (TAK1) is a quintessential kinase that positively regulates inflammation through NF-κB and MAPK signaling cascades. However, the effects of TAK1 on neuroinflammation in EBI following SAH are largely unknown.

**Methods:**

Two hundred and forty-six male C57BL/6J mice were subjected to the endovascular perforation model of SAH. A selective TAK1 inhibitor, 5Z-7-oxozeaenol (OZ) was administered by intracerebroventricular (i.c.v) injection at 30 min after SAH induction. To genetic knockdown of TAK1, small interfering RNA (siRNA) was i.c.v injected at 48 h before SAH induction. SAH grade, brain water content, BBB permeability, neurological score, western blot, real-time PCR, ELISA, transmission electron microscope, and immunofluorescence staining were performed. Long-term behavioral sequelae were evaluated by the rotarod and Morris water maze tests. Furthermore, OZ was added to the culture medium with oxyhemoglobin (OxyHb) to mimic SAH in vitro. The reactive oxygen species level was detected by DCFH-DA staining. Lysosomal integrity was assessed by Lyso-Tracker Red staining and Acridine Orange staining.

**Results:**

The neuronal phosphorylated TAK1 expression was upregulated following SAH. Pharmacologic inhibition of TAK1 with OZ could alleviate neurological deficits, brain edema, and brain-blood barrier (BBB) disruption at 24 h after SAH. In addition, OZ administration restored long-term neurobehavioral function. Furthermore, blockade of TAK1 dampened neuronal pyroptosis by downregulating the N-terminal fragment of GSDMD (GSDMD-N) expression and IL-1β/IL-18 production. Mechanistically, both in vivo and in vitro, we demonstrated that TAK1 can induce neuronal pyroptosis through promoting nuclear translocation of NF-κB p65 and activating nucleotide-binding oligomerization domain (NOD)-like receptor pyrin domain containing 3 (NLRP3) inflammasome. TAK1 siRNA treatment mitigated SAH-induced neurobehavioral deficits and restrained phosphorylated NF-κB p65 expression and NLRP3 inflammasome activation. TAK1 blockade also ameliorated reactive oxygen species (ROS) production and prevented lysosomal cathepsin B releasing into the cytoplasm.

**Conclusions:**

Our findings demonstrate that TAK1 modulates NLRP3-mediated neuronal pyroptosis in EBI following SAH. Inhibition of TAK1 may serve as a potential candidate to relieve neuroinflammatory responses triggered by SAH.

**Supplementary Information:**

The online version contains supplementary material available at 10.1186/s12974-021-02226-8.

## Introduction

Aneurysmal subarachnoid hemorrhage (SAH) is a devastating form of cerebral vascular disease with significant patient disability and mortality [1]. Early brain injury (EBI), which occurs in the brain before the onset of delayed vasospasm, may contribute to poor outcomes following SAH [1, 2]. Transient global ischemia and toxicity of subarachnoid blood initiate excessive innate immune response during the EBI period, causing secondary injury to the brain [3]. Accumulating evidences suggested that neuronal cell is involved in immune responses in ischemic stroke, such as nucleotide-binding oligomerization domain (NOD)-like receptor pyrin domain containing 3 (NLRP3) inflammasome-mediated inflammation [4, 5]. However, the cellular mechanisms responsible for neuronal inflammation following SAH remain to be fully understood. Targeting neuronal inflammation reaction may be helpful for search therapeutic targets for SAH.

Transforming growth factor-β-activated kinase 1 (TAK1) belongs to the mitogen-activated protein kinase kinase kinase (MAP3K) family and functions as a critical signaling molecule in innate immune signaling pathways activated by cytokines and Toll-like receptors [6]. TAK1 is expressed in neurons and pharmacological blockade of TAK1 could inhibit the activation of mitogen-activated protein kinases (MAPKs) and nuclear factor-κB (NF-κB) signaling cascades following SAH in rats [7]. TAK1 is reported as a downstream factor of TRAF3, GPR120, and CaMKII in the pathophysiological process of EBI after SAH [8–10]. Previous studies indicated that TAK1 regulates lysosomal rupture-induced and altering cellular volume-induced NLRP3 inflammasome activation [11, 12]. NLRP3 inflammasome activation converts precursor caspase-1 into cleaved caspase-1, which further cleaves precursors interleukin (IL)-1β and IL-18 into biologically active mature proinflammatory cytokines and cleaves gasdermin D (GSDMD) to trigger pyroptosis [13, 14]. In addition, inhibition of TAK1 could elicit caspase-8-dependent macrophage pyroptosis [15]. To date, no study reported the effects of TAK1 on neuronal pyroptosis in SAH models.

Herein, using the mouse SAH model and oxyhemoglobin-treated neurons, we sought to investigate the role of TAK1 in SAH and whether TAK1 inhibition could prevent EBI by reducing NLRP3 inflammasome-associated pyroptosis in neurons.

## Methods and materials

### Animals

The Animal Ethics Review Committee of The First Affiliated Hospital of the University of Science and Technology of China approved all the procedures. The study was implemented according to the National Institute of Health Guide for the Care and Use of Laboratory Animals (NIH Publications No. 80-23, revised 1996). A total of 246 C57BL/6J male mice (18–22 g) were used in this study. Mice were housed in laboratory cages at 22–24 °C and 55–60 % humidity, with a 12-h light/dark cycle and free access to food and water.

### Study protocol

All mice were randomly divided into five separate experiments (Supplementary Fig. S1).

#### Experiment 1

Mice were randomly divided into 6 groups (Sham, 2 h, 6 h, 12 h, 24 h, and 72 h after SAH, *n* = 5 for each group). Western blot analysis was performed to detect the expression of phosphorylated TAK1(p-TAK1) and TAK1 in the ipsilateral cortex of mice after SAH. Additional 4 mice from sham (*n* = 2) and SAH 24h (*n* = 2) group were used for double immunostaining.

#### Experiment 2

To elucidate the effect of TAK1 on EBI, TAK1 inhibitor 5Z-7-oxozeaenol (OZ, Sigma-Aldrich, USA) was used. Sixty-four mice were randomly divided into the Sham (*n* = 16), SAH+Vehicle (DMSO, *n* = 16), SAH+1μg OZ (*n* = 16), and SAH+3μg OZ (*n* = 16) groups. The drug was intracerebroventricular administrated at 30 min post-SAH. Neurological scores, SAH severity, brain water content, IgG staining, Fluoro-Jade C (FJC) staining, and the expression of tight junction proteins were evaluated 24 h post-modeling.

#### Experiment 3

Based on the results of neurological performance, a 3-μg dosage of OZ was used to determine the effect of TAK1 on long-term neurological functional recovery following SAH. Thirty mice were randomly assigned into three groups: Sham (*n* = 10), SAH+Vehicle (DMSO, *n* = 10), SAH+3μg OZ (*n* = 10). A rotarod test was performed on day 7, day 14, and day 21 after SAH. The Morris water maze test was performed on days 22–28 after SAH.

#### Experiment 4

To explore the potential mechanisms of TAK1 on neuroinflammation, forty-five mice were randomly divided into the Sham (*n* = 15), SAH+Vehicle (DMSO, *n* = 15), and SAH+3 μg OZ (*n* = 15) groups. Assessment methods including double immunostaining, transmission electron microscope, western blot, real-time PCR, and enzyme-linked immunosorbent assay (ELISA).

#### Experiment 5

To investigate the effect of genetic inhibition for TAK1 on neuroinflammation, TAK1 small interfering RNA(TAK1 siRNA) was administered intracerebroventricularly at 48 h before SAH induction. Mice were randomly assigned to four groups: Sham (*n* = 10), SAH+Vehicle (DMSO, *n* = 10), SAH+Scrambled siRNA(Scr siRNA, *n* = 10), and SAH+TAK1 siRNA(*n* = 10). Western blot and NeuN/Cy5-conjugated TAK1 siRNA double immunostaining were performed to validate the knockdown efficiency of TAK1 siRNA. Neurological scores, brain water content, and Western bolt were assessed at 24 h after SAH.

### SAH modeling

The endovascular filament perforation SAH model was produced as described previously [16, 17]. Briefly, the mice were anesthetized with 1% pentobarbital; then, the right internal carotid artery (ICA) was dissected from the adjacent tissue. A sharpened monofilament nylon (diameter: 0.18 ± 0.01 mm, Beijing Cinontech Co., Ltd, China) was used to puncture the bifurcation of the right middle cerebral artery and the anterior cerebral artery through the ICA. Sham mice underwent the same procedure except that the suture was only advanced 3 mm into the right ICA without perforating the artery. During the operation, body temperature was maintained at 37 °C ± 0.5 °C with a heating pad. Physiological parameters, including arterial blood gases (pH, P_CO2_, and P_O2_), mean blood pressure (MABP), and plasma glucose, were monitored according to previous methods [18].

### Intracerebroventricular injection

The intracerebroventricular (i.c.v) drug administration was performed as previously described [19]. Briefly, mice were placed in a stereotaxic frame after intraperitoneal anesthesia with 1% pentobarbital. A 10-μl Hamilton syringe (Shanghai Gaoge Industry & Trade Co., Ltd., China) was inserted into the right lateral ventricle at the following coordinates: 0.4 mm posterior and 1.0 mm lateral to the bregma, and 3.0 mm below the dural layer. Two microliters of DMSO or OZ (1 or 3μg; Sigma-Aldrich, USA) dissolved in 2 μl DMSO was infused at 30 min post-SAH. 2′OMe+5′Chol+5′Cy5 modified TAK1 short interfering RNA (siRNA) and scrambled siRNA were purchased from RiboBio (Guangzhou, China) and then prepared at a concentration of 500 p.m./μL in RNase free resuspension buffer. A total volume of 3.0 μL TAK1 siRNA (sense: 5′-GGUCUGUUAUACCAAAUAATT-3′; antisense: 3′-AGCCAGACAAU AUGGUUUAUU-5′ ) or scrambled siRNA was injected at 48 h before SAH induction. OZ or siRNA was injected at the rate of 0.5 μl/min by a pump.

### SAH grade

The severity of SAH was blindly assessed at 24 h post-modeling [20]. The basal cistern was divided into six segments, and each segment was scored from 0 to 3 depending on the amount of subarachnoid blood clot. The total score ranged from 0 to 18 adding the scores from all 6 segments.

### Brain edema measurement

Brain edema was determined according to our previous methods [21]. Twenty-four hours after SAH, mice brains were harvested and divided into ipsilateral and contralateral hemispheres. The tissues were weighed immediately to obtain the wet weight and again after drying in an oven at 105 °C for 24 h to obtain dry weight. The percentage of water content was calculated according to the following formula: ([wet weight-dry weight]/wet weight) × 100%.

### Behavioral analysis

The short-term neurological performance was blindly evaluated using the modified Garcia score test and beam balance test as previously described [22]. The modified Garcia score is an 18-point scoring system, including spontaneous activity (0–3), symmetry in the movement of four limbs (0–3), forepaw outstretching (0–3), climbing (1–3), body proprioception (1–3), and response to vibrissae touch (1–3), in which higher scores indicated better function. To performed the beam balance test, mice were placed on a 1-m beam with a flat surface of 6-mm width, and each mouse was allotted a score of 0 to 3 according to the walking distance with 1 min.

The long-term neurological performance was assessed by the rotarod test and the Morris water maze test. For the rotarod test [23], mice were placed on a rotarod cylinder (RWD, China). The rotating speed was slowly accelerated from 0 revolutions per minute (RPM) to 30 RPM by 3 RPM every 10 s. The time remaining on the rotarod was recorded. For the Morris water maze test [24], mice were individually trained in a circular pool (100-cm diameter, 60-cm height) filled with water (22 °C). The platform was hidden 1 cm below the surface of the water. Mice were trained 4 times from different quadrants per day for 5 consecutive days to accomplish hidden platform training. Each trial lasted either until the mice found the platform or for 90 s. Twenty-four hours after the last training, the platform was moved and mice were subjected to explore the platform for 60 s. Length of swim path, time to reach the platform (latency), time spent in the target quadrant, and number of times animals crossed above the former target site where the platform had been located (crossovers) were recorded by the ANY-maze video tracking software (Stoelting, USA). The observer and recorder were blinded to animal grouping.

### Histological staining

Immunofluorescence staining was performed according to our previous study [24]. Mice were euthanized at 24 h post-modeling and intracardially perfused with PBS followed by 4% paraformaldehyde (PFA). Brains were postfixed in 4% PFA for 4 h, then dehydrated in gradient sucrose solutions of 10%, 20%, and 30% for 24 h each. Brains were embedded and frozen in Tissue-Tek O.C.T. compound (Sakura Finetek, USA). Fifteen micrometers of brain coronal sections were cut at 1.0 mm posterior to the bregma with a Leica CM1950 cryostat. Sections were incubated with primary antibodies overnight at 4 °C. The following antibodies were used: rabbit anti-phospho-TAK1(Thr184/187) (p-TAK1; 1:1000, MA5-15073, Thermo Fisher Scientific, USA), rabbit anti-NeuN (1:500; ab177487, Abcam, UK), mouse anti-NeuN (1:500; ab104225, Abcam, UK), and mouse anti-GSDMD (1:200; sc-393581, Santa Cruz Biotechnology, USA). After three washes in PBS, tissue samples were incubated for 2 h with the following secondary antibodies: AlexaFluor 488 goat anti-rabbit IgG, AlexaFluor 594 donkey anti-mouse IgG, AlexaFluor 488 donkey anti-mouse IgG, or AlexaFluor 594 donkey anti-rabbit IgG (Jackson, USA), followed by counterstaining with DAPI for 10 min. Pictures were acquired with an Olympus FV3000 microscope (Olympus, Japan).

For IgG staining, brain slices were incubated with biotin-conjugated donkey anti-mouse IgG antibody (1:200; Jackson, USA) for 2 h at room temperature. Staining was revealed using an ABC kit (Beyotime Biotechnology, China). To detect degenerated neurons, FJC (Millipore, USA) staining was performed as previously described [25]. Frozen slides were sequentially immersed in 1% sodium hydroxide solution, 70% ethanol, and 0.06% potassium permanganate solution. Then, the sections were incubated with 0.0001% solution of FJC. The regions of interest selected for image acquisition and quantitative analysis are depicted in Supplementary Fig. S2. The positive cells were counted and analyzed using the Image J software (NIH, USA) by a blinded observer.

### Transmission electron microscopy

Brain tissues were cut into 1 mm^3^ and fixed in 2.5% glutaraldehyde for 24 h, followed by post-fixation in 1% osmium tetroxide for 3 h. After being dehydrated in gradient ethanol (50%, 70%, 90%, and 100%) for 10 min each, the samples were embedded in resin and cut into a thickness of 50–60 nm. The sections were stained with uranyl acetate and lead citrate and examined under a transmission electron microscope (HT7700, Hitachi, Japan).

### Cell culture and in vitro model

Primary neurons were cultured according to our previous methods [24]. Briefly, cortical brain tissues from fetal mice (E14) were collected and incubated with 0.125% trypsin at 37 °C for 15 min. After termination with 10% FBS/DMEM, the supernatant of tissue mixture was filtered using a 100-μm cell strainer. The cells were resuspended in 10% FBS/DMEM and seeded on poly-l-lysine-coated plates. After 2 h, the cultured medium was replaced with a neurobasal medium containing 2% B27 and 1% Glutamax and renewed every 3 days.

Oxyhemoglobin (OxyHb) was produced using mouse hemoglobin (Sigma-Aldrich, USA) according to the manufacturer’s instructions. To mimic SAH in vitro, primary cultured neurons were incubated with OxyHb at a concentration of 25 μM according to a previous report [26]. For in vitro TAK1 inhibition assay, neurons were pretreated with OZ (600 nM) for 2 h before being exposed to OyxHb [27]. To mimic cellular ROS accumulation, l-buthionine-sulfoximine (BSO, Sigma-Aldrich, USA) was premixed with a culture medium at a concentration of 1 mM before the in vitro SAH model was induced [28]. To explore the neurotoxicity of IL-1β, primary neurons were treated with 10 ng/ml mouse IL-1β recombinant protein (rIL-1β; Thermo Fisher Scientific, USA) for 24 h [29].

### ROS detection

The production of superoxide anions of brain tissue was investigated with dihydroethidium (DHE, Thermo Fisher Scientific, USA) staining. In brief, freshly brain sections were incubated with 1 μM DHE for 10 min at room tempreture, followed by stained with DAPI for 5 min. To detect the reactive oxygen species (ROS) level in neurons, 2′,7′-dichlorofluorescin diacetate (DCFH-DA; 5μM, Sigma-Aldrich, USA) was added to the neurobasal medium and incubated for 20 min. The fluorescence density was quantified with the Image J software (NIH, USA). The concentration of total SOD in brain tissues or neurons was detected by an SOD assay kit (Nanjing Jiancheng Bioengineering Institute, Nanjing, China).

### Live-cell staining

OZ-primed neurons were incubated with OxyHb for 24 h; then, cells were washed twice with DMEM. To assess the lysosome, cells were incubated with Lyso-Tracker Red (100 nM) for 30 min, subsequently incubated with Hochest 33342 (20 μg/ml) for 10 min. For acridine orange staining, primary neurons were incubated with 2 μg/ml of acridine orange for 30 min. Then, the signal was analyzed using a fluorescence microplate reader according to previous methods [30]. Images were captured by an Olympus FV3000 microscope (Olympus, Japan).

### ELISA

The protein concentrations of IL-1β and IL-18 in the supernatant of brain tissue homogenate or cell culture were measured using ELISA kits (SMLB00C and #7625, R&D systems, USA) according to the manufacturer’s instructions.

### Real-time polymerase chain reaction

Total RNA was extracted from primary neurons and ipsilateral cortex tissue using TRIzol Reagent (Takara, Japan). Five hundred nanograms of total RNA was reverse transcribed into cDNA with the RevertAid First Strand cDNA Synthesis Kit (Thermo Fisher Scientific, USA). Then, real-time PCR was performed with UltraSYBR Mixture (CWBio, China) using the Mx3000P Real-Time PCR System (Agilent Technologies, USA) under the following conditions: predenaturation at 95 °C for 10 min, followed by 40 cycles at 95 °C for 15 s, 60 °C for 1min. Primers used in the study are listed in Table 1. The levels of mRNA were normalized in relevance to GAPDH.

**Table 1:** Primers used in real-time PCR

Gene	Sense primer (5′-3′)	Antisense primer (3′-5′)
NLRP3	GCATTGCTTCGTAGATAGAGG	GATGAAGGACCCACAGTGTAA
IL-1β	TTGTTCATCTCGGAGCCTGTA	AGCACCTTCTTTTCCTTCATC
IL-18	ACCACTTTGGCAGACTTCACT	ACACAGGCGGGTTTCTTTTG
GAPDH	AAGAAGGTGGTGAAGCAGG	GAAGGTGGAAGAGTGGGAGT

### Western blotting

Total protein and nuclear fractions were prepared using RIPA lysis buffer (Cell Signaling Technology, USA) and NE-PER™ Nuclear and Cytoplasmic Extraction Reagents (Thermo Fisher Scientific, USA) respectively. Lysosome was extracted using Lysosome Isolation Kit (LYSISO1-1KT, Sigma-Aldrich, USA). Equal amounts of protein were loaded and separated on 6–10% SDS-PAGE gel, then electrophoresed and transferred to polyvinylidene difluoride membranes (Millipore, USA). The membranes were blocked with 5% non-fat milk and incubated overnight at 4 °C with primary antibodies against p-TAK1(Thr184/187) (1:1000, MA5-15073, Thermo Fisher Scientific, USA), TAK1 (1:1000; #5206, Cell Signaling Technology, USA), ZO-1 (1:1000; ab276131, Abcam, UK), Occludin (1:1000; ab216327, Abcam, UK), NLRP3 (1:500; sc-66846, Santa Cruz Biotechnology, USA), ASC (1:500; sc-33958, Santa Cruz Biotechnology, USA ), Caspase-1 (1:1000; #3866, Cell Signaling Technology, USA), IL-1β (1:2000; ab9722, Abcam, UK), IL-18 (1:1000; ab71495, Abcam, UK), GSDMD (1:1000; ab219800, Abcam, UK), phospho-IκBа (1:1000; #2859, Cell Signaling Technology, USA), IκBа (1:1000; #9242, Cell Signaling Technology, USA), phospho-NF-κB p65 (1:1000; #3033, Cell Signaling Technology, USA), NF-κB p65 (1:1000; #8242, Cell Signaling Technology, USA), Cathepsin B (1:100; sc-365558, Santa Cruz Biotechnology, USA ), LAMP1 (1:1000; ab24170, Abcam, UK), Histone H3 (1:1000; #4499, Cell Signaling Technology, USA) and β-actin (1:1000; #4970, Cell Signaling Technology, USA). After being washed with TBST three times, membranes were incubated with HRP-conjugated secondary antibody for 1 h. Protein signal was detected by Immobilon Western Chemiluminescent HRP Substrate (Millipore, USA) and quantified using the Image J software (NIH, USA).

### Statistical analysis

The SPSS 22.0 software (IBM, Armonk, NY, USA) was used for the statistical analysis. All data were expressed as the mean and standard deviation (mean ± SD). Student’s *t-*test was used to compare the two groups, and one-way ANOVA followed by Tukey’s post hoc test was used to compare more than two groups. For the data of escape latency and swimming path length, two-way repeated-measures ANOVA followed by Tukey’s post hoc test was performed. A *P* value of < 0.05 was considered statistically significant.

## Results

### SAH mortality and severity

The overall mortality of SAH mice was 14.36%. Seven mice were excluded from the study due to mild SAH grades ≤ 8. No significant difference was found in SAH mortality and severity among experimental groups (Supplementary Fig. S3). In addition, physiological parameters (pH, P_CO2_, P_O2_, MABP, and plasma glucose) were similar among the groups (Supplementary Table S1).

### Expression pattern of TAK1 in the ipsilateral cortex after SAH

**Fig. 1:**
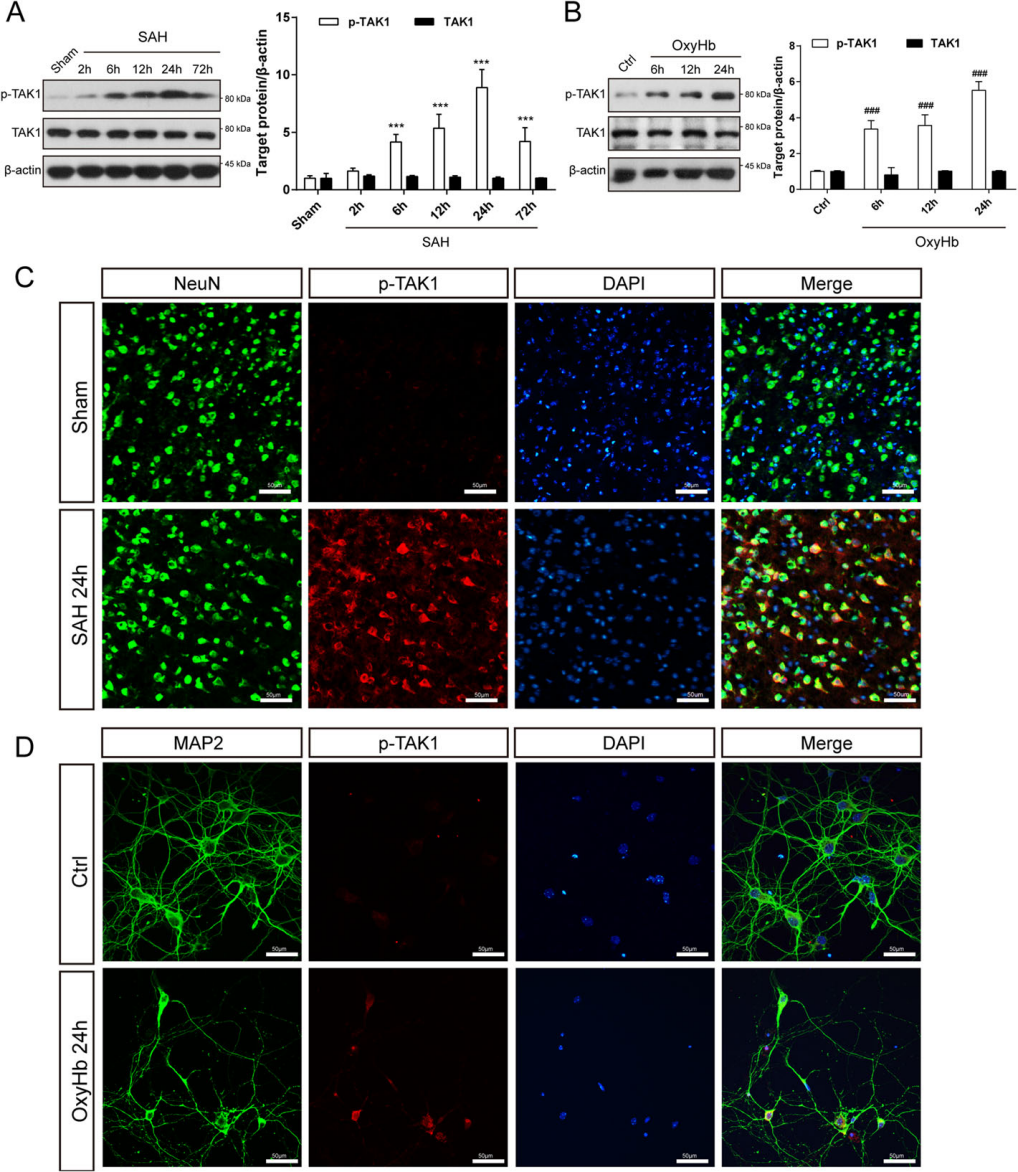
Temporal expression of TAK1 in the ipsilateral cortex following SAH. **a** Western blot showing p-TAK1 and TAK1 expression at 2 h, 6 h, 12 h, 24 h, and 72 h following SAH onset (*n* = 5 per group). **b** Western blot assay for the expression of p-TAK1 and TAK1 in OxyHb-exposed neurons (*n* = 5 per group). **c** Co-staining of p-TAK1 (red) and NeuN (green) demonstrated that p-TAK1 was upregulated in neurons 24 h after SAH (*n* = 2 per group). **d** Representative immunofluorescence staining images of p-TAK1 in control and OxyHb-exposed groups (*n* = 5 per group). Data are expressed as mean ± SD. ***P < 0.001 vs Sham group, ^###^*P* < 0.001 vs Control group. Scale bar: 50 μm

To determine whether TAK1 is activated after SAH, we detected the protein levels of p-TAK1 (Thr184/187) and TAK1 by western blot. As shown in Fig. 1a, p-TAK1 was triggered at 6 h post-SAH, plateaued at 24 h post-SAH, and then dropped at 72 h post-SAH. However, the expression of total TAK1 did not change among groups. To mimic the conditions of SAH, primary cortical neurons were stimulated by OxyHb. Western blot results showed that the expression level of p-TAK1(Thr184/187) in neurons was significantly increased after incubated with OxyHb, and continuously increased as the exposure time prolongs (Fig. 1b). Double immunofluorescence labeling of p-TAK1 and NeuN demonstrated that the number of p-TAK1 positive neurons was increased in SAH mice (24 h) compared to that in sham mice (Fig. 1c). Similarly, OxyHb incubation enhanced the p-TAK1 immunostaining signal in primary neurons (Fig. 1d).

### OZ treatment ameliorates neurological deficits and BBB disruption after SAH

**Fig. 2:**
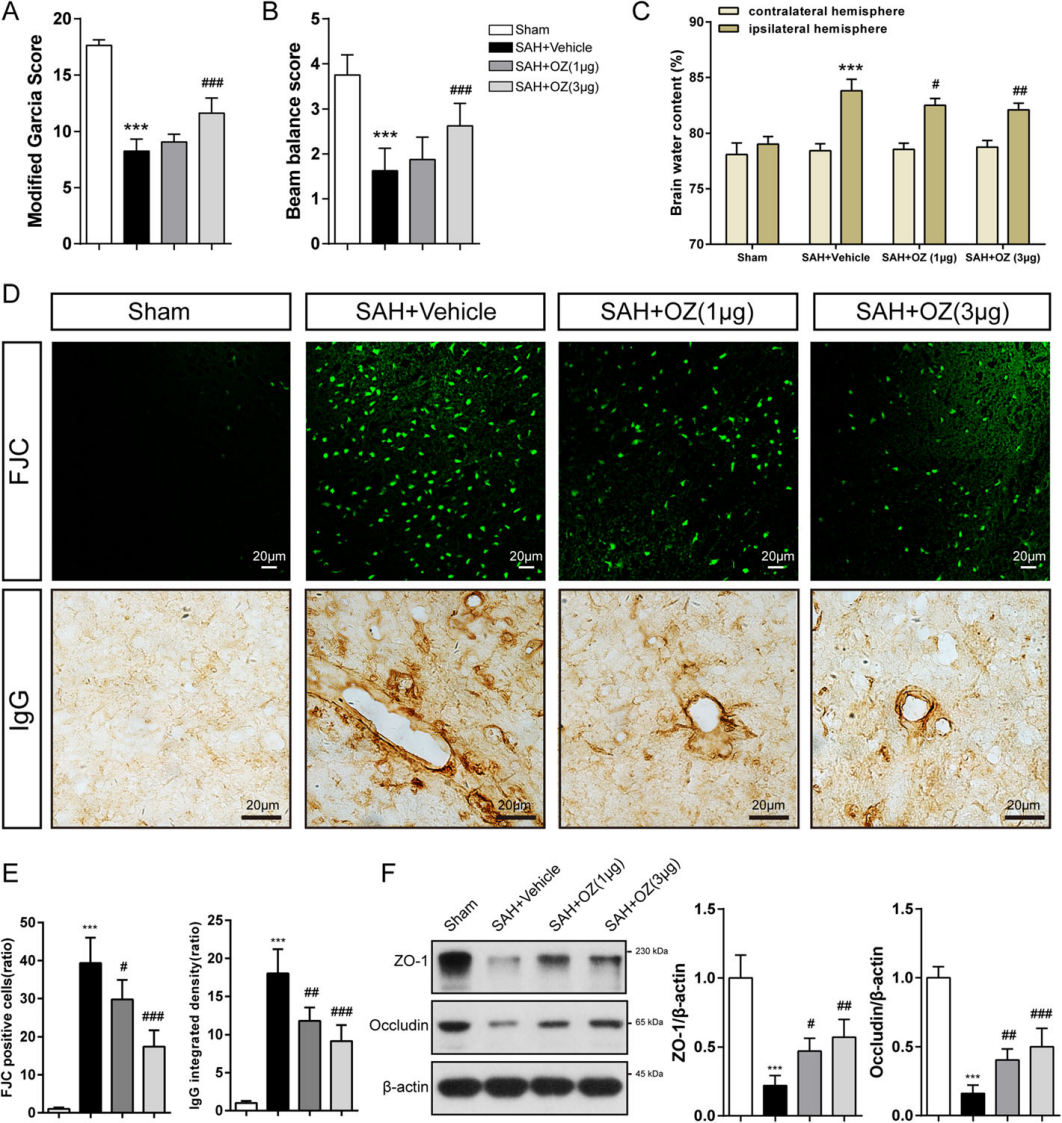
TAK1 inhibition improves neurological functions and attenuates brain edema 24h after SAH. **a** Modified Garcia score, **b** Beam balance test, and **c** brain water content in Sham, SAH+Vehicle, SAH+OZ (1 μg), and SAH+OZ (3 μg) groups at 24 h after SAH. **d**, **e** FJC staining and IgG staining in the ipsilateral cortex with quantification. **f** Immunoanalysis of tight junction proteins (ZO-1 and Occludin) in brain extracts of the indicated groups. The right panels show the quantification of the proteins. In **a** and **b**, n = 16 for each group. In **c**, n = 6 for each group. In **d** and **f**, n = 5 in each group. Data are expressed as mean ± SD. ***P < 0.001 vs Sham group; ^#^*P* < 0.05, ^##^*P* < 0.01, ^###^*P* < 0.001 vs SAH+Vehicle group. Scale bar: 20 μm

To assess the role of TAK1 in EBI after SAH, OZ, a specific inhibitor of TAK1 was administrated. OZ (1 μg or 3 μg) treatment significantly reduced the protein levels of p-TAK1 and TAK1 compared to the vehicle-treated SAH mice (Supplementary Fig. S4). Remarkable neurological impairment was observed in the vehicle-treated group compared with that in the sham group at 24 h after SAH as evaluated by modified Garcia score and beam balance test (Fig. 2a, b, both *P* < 0.001). Administration of 1 μg of OZ did not improve neurological performance, whereas 3 μg of OZ significantly improved neurological function (Fig. 2a, b, both *P* < 0.001). The brain water content of the ipsilateral hemisphere notably increased in the vehicle-treated SAH mice, which was substantially mitigated by treatment with both dosages of OZ (Fig. 2c, *P* = 0.025 and *P* = 0.002). We then tested whether OZ could rescue neuronal degeneration. The images displayed that FJC-positive neurons after SAH were significantly decreased by inhibition of TAK1 (Fig. 2d, e, *P* = 0.026 and *P* < 0.001). Pharmacological blockade of TAK1 inhibited SAH-induced IgG extravasation in the ipsilateral cortex (Fig. 2d, e, *P* = 0.0012 and *P* < 0.001). Tight junction proteins (ZO-1 and Occludin) were also detected to further evaluate BBB permeability. Reduced tight junction proteins expression were observed in SAH+Vehicle mice, while OZ application markedly preserved tight junction proteins (Fig. 2f, for ZO-1: *P* = 0.0213, *P* = 0.0014, for Occludin: *P* = 0.0033 and *P* = 0.0001). Based on these findings, OZ at the dosage of 3 μg was selected for the following experiments.

### TAK1 inhibition improves long-term neurobehavioral function

**Fig. 3:**
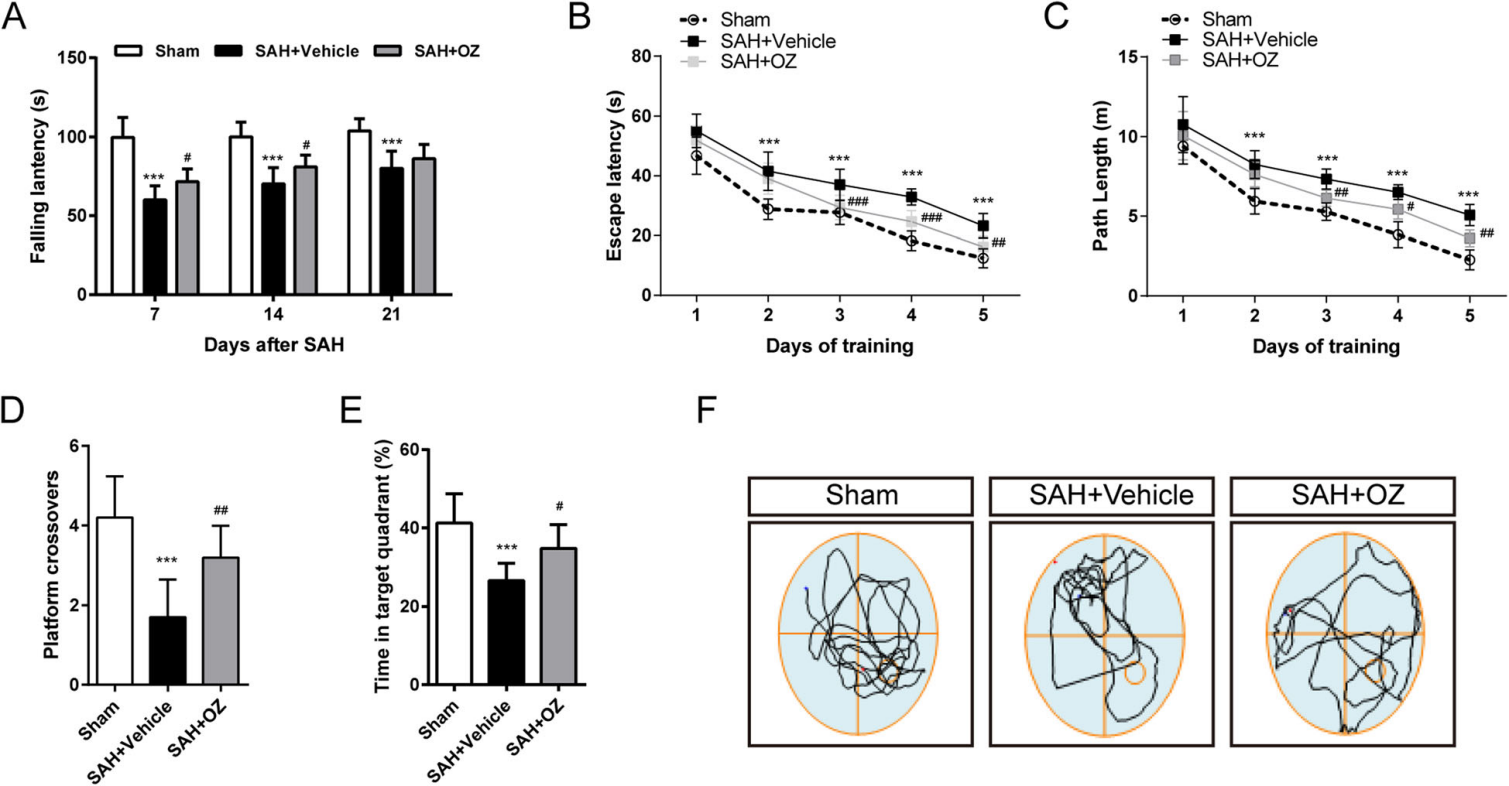
TAK1 inhibition improves long-term neurological function after SAH. **a** Rotarod test of mice at days 7, 14, and 21 after SAH. **b** Escape latency and **c** swimming distance of the Morris water maze test in Sham, SAH+Vehicle, and SAH+OZ groups. **d** Platform crossovers and **e** percentage of time in the target quadrant in the probe trial. **f** Representative swimming trajectories of the three groups in the probe trial. Data are expressed as mean ± SD, n = 10 per group. ***P < 0.001 vs Sham group; ^#^*P* < 0.05, ^##^*P* < 0.01, ^###^*P* < 0.001 vs SAH+Vehicle group

Vehicle-treated SAH mice showed shorter falling latency in the rotarod test compared with the sham-operated mice on days 7, 14, and 21 after SAH. However, OZ treatment significantly improved the rotarod performance on days 7 and 14 post-SAH (Fig. 3a, *P* = 0.0232 and *P* = 0.0375).

Cognitive testing was evaluated among groups by the Morris water maze. During the acquisition of spatial learning, SAH+Vehicle mice spent more time and longer swimming distance to locate the hidden platform compared with controls, whereas OZ treatment improved spatial learning by significantly shortening escape latency and swimming distance on days 3, 4, and 5 (Fig. 3b, c, for escape latency: *P* < 0.001, *P* < 0.001 and *P* = 0.002, for swimming distance: *P* = 0.0085, *P* = 0.0207 and *P* = 0.0011). Moreover, in the probe phase, OZ application significantly increased the crossovers of the previous platform location and the time spent in the target quadrant (Fig. 3d, e, *P* = 0.0034 and *P* = 0.0159).

### TAK1 activation triggers neuronal pyroptosis after SAH

**Fig. 4:**
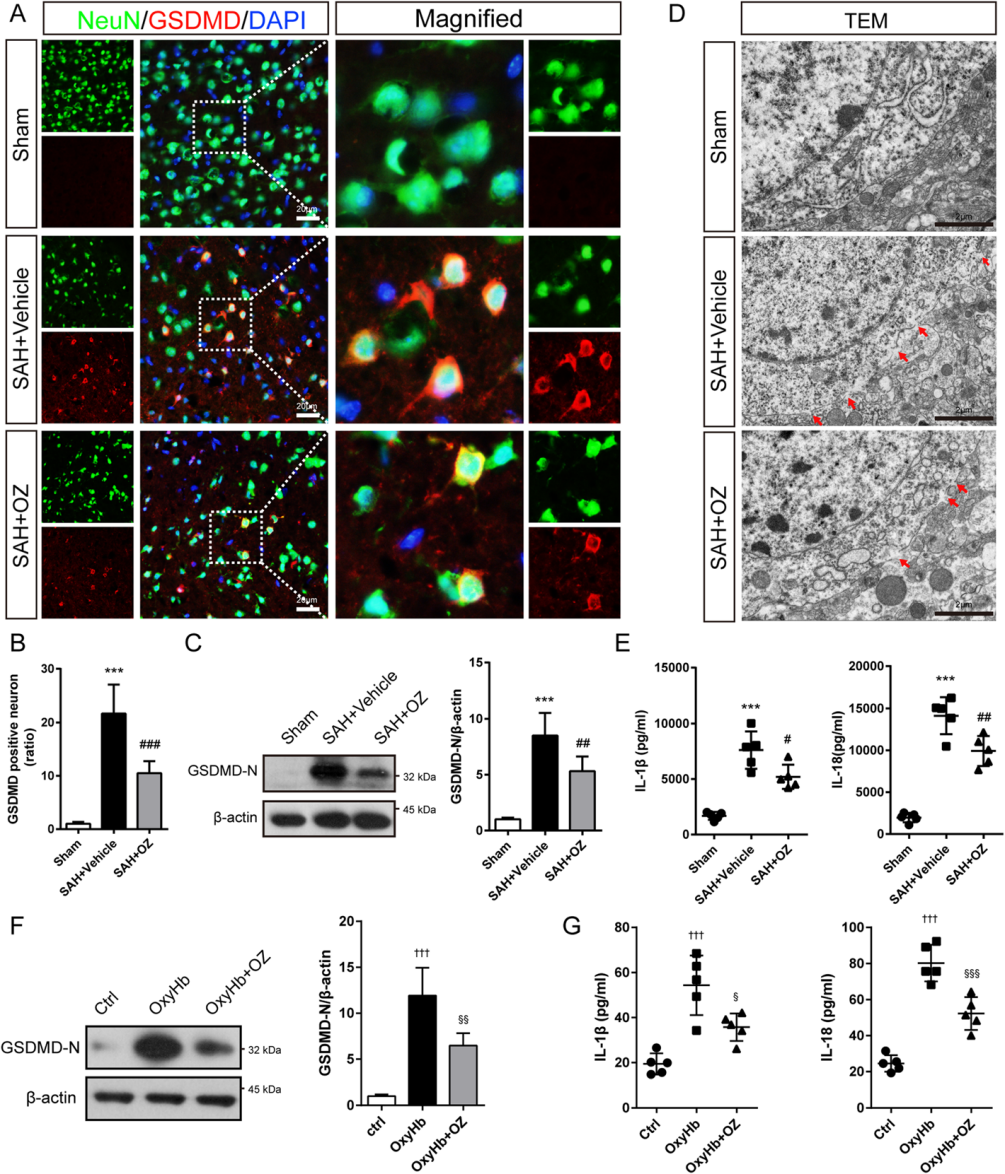
OZ treatment reduces neuronal pyroptosis after SAH. **a** Double immunostaining of NeuN and GSDMD and **b** quantitative analysis of GSDMD-positive neurons in ipsilateral cortex 24 h after SAH. Scale bar: 20 μm. **c** Immunoblot analysis of GSDMD expression in the treated mice. **d** Representative transmission electron micrographs of neurons in brain tissues. Red arrowhead: membrane pores. Scale bar: 2 μm. **e** The levels of IL-β and IL-18 in the ipsilateral cortex were analyzed by ELISA kits. **f** Western blot and quantitative analysis of GSDMD in the Control, OxyHb, and OxyHb+OZ groups. **g** The concentration of IL-1β and IL-18 in the supernatant of the cell culture. Data are expressed as mean ± SD, *n* = 5 per group. ***P < 0.001 vs Sham group; ^#^*P* < 0.05, ^##^*P* < 0.01, ^###^*P* < 0.001 vs SAH+Vehicle group. ^†††^*P* < 0.001 vs Control group; ^§^*P* < 0.05, ^§§^*P* < 0.01, ^§§§^*P* < 0.001 vs OxyHb group

Next, we investigated whether neuronal pyroptosis is affected by TAK1 activation post-SAH. Intense GSDMD immunostaining concentrated at the neuronal membrane was observed in SAH mice, forming a “ring of fire” morphology (Fig. 4a). Relative to the sham group, SAH induced a 21.7-fold increase in GSDMD-positive neurons in the ipsilateral cortex. However, this trend was reversed with OZ treatment (Fig. 4a, b, *P* < 0.001). Western blot analysis revealed that the protein levels of the GSDMD-N terminal (GSDMD-N), the activated form of GSDMD, were obviously increased in SAH mice compared to those of the sham group. Yet, administration of OZ significantly prevented the post-SAH increase in GSDMD-N expression (Fig. 4c, *P* = 0.0090). Pyroptosis is characterized by membrane pores formation. To observe the changes in the neuronal cell membrane post-SAH, a transmission electron microscope was used. As shown in Fig. 4d, the GSDMD membrane pore in neurons at the ipsilateral cortex region was increased 24 h after SAH compared with sham mice; however, OZ injection mitigated this trend. The ELISA assay showed the levels of IL-1β and IL-18 in the ipsilateral cortex of SAH mice were also reduced after treatment with OZ (Fig. 4e, *P* = 0.0183 and *P* = 0.0048).

In vitro, primary neurons were incubated with 600 nM of OZ to inhibit TAK1. As shown in Supplementary Fig. S5, OZ pre-treatment significantly reduced the protein levels of p-TAK1 and TAK1. The enhanced GSDMD-N level in the OxyHb group was strikingly attenuated by pre-treatment with OZ (Fig. 4f, *P* = 0.0023). Besides, OxyHb treatment significantly upregulated the levels of IL-1β and IL-18 in the cell culture supernatant, and these increments were pronouncedly reduced by pre-treatment with OZ (Fig. 4g, *P* = 0.0154 and *P* < 0.001). These results indicate that OZ administration attenuates OxyHb-induced neuronal pyroptosis. Furthermore, we found that mouse rIL-1β could trigger the protein expression of p-TAK1, NLRP3, ASC, and Caspase-1 in primary neurons (Supplementary Fig. S6).

### NLRP3 inflammasome activation is involved in TAK1-induced neuronal pyroptosis

**Fig. 5:**
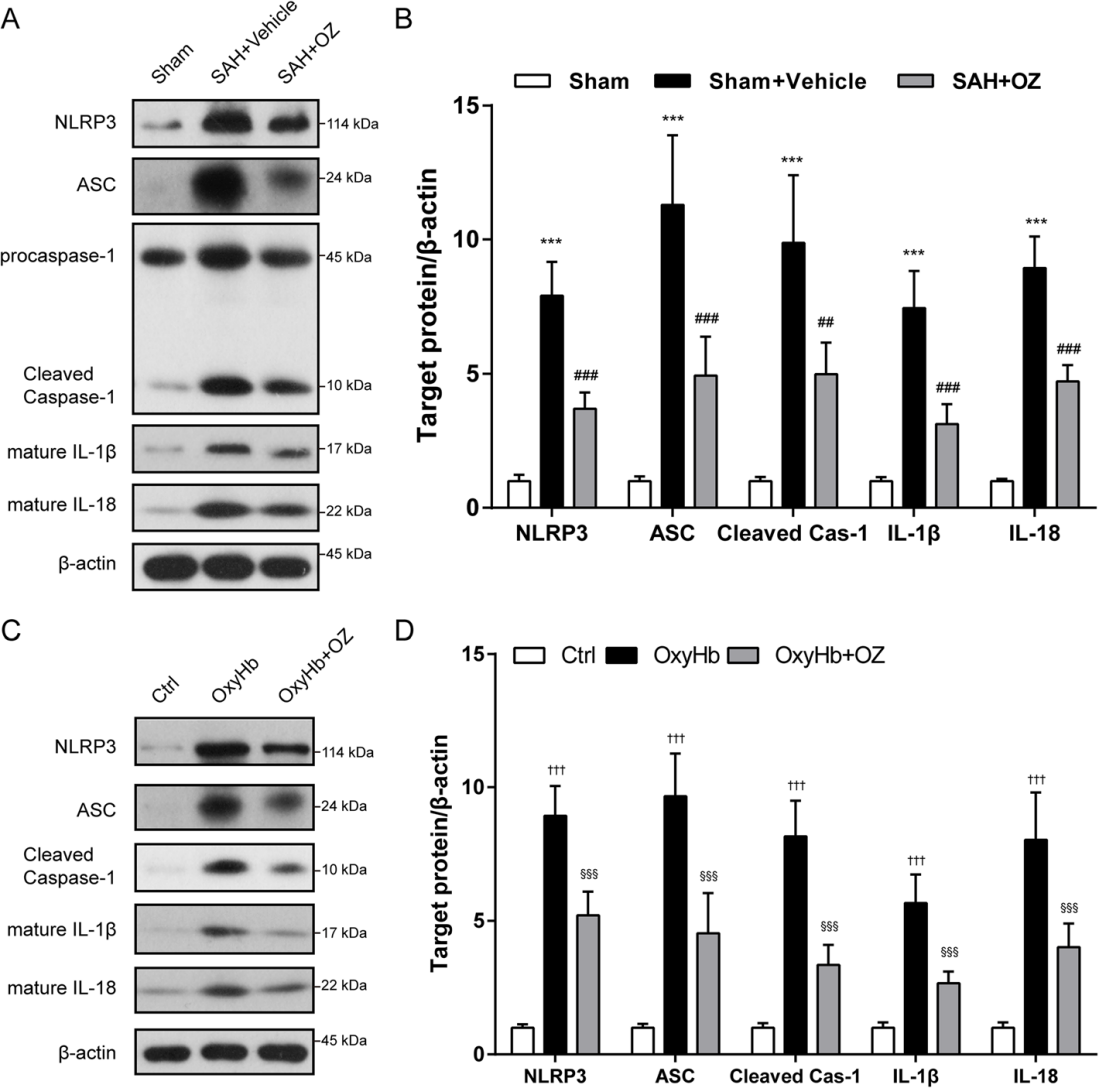
Blockade of TAK1 prevents neuronal pyroptosis through activating NLRP3 inflammasome. **a** Representative immunoblot images and **b** quantification of NLRP3, ASC, cleaved caspase-1, IL-β, and IL-18 in the ipsilateral cortex 24 h after SAH. **c** Western blotting and **d** quantitative analysis of NLRP3, ASC, cleaved caspase-1, IL-β, and IL-18 in Ctrl-, OxyHb-, and OxyHb+OZ-treated neurons. Data are expressed as mean ± SD, *n* = 5 per group. ***P < 0.001 vs Sham group; ^##^*P* < 0.01, ^###^*P* < 0.001 vs SAH+Vehicle group. ^†††^*P* < 0.001 vs Control group;^§§§^*P* < 0.001 vs OxyHb group

Pyroptosis is a lytic form of regulated cell death that relies on cytosolic inflammasome activation [14]. To this end, we tested whether TAK1 triggers neuronal pyroptosis through activating NLRP3 inflammasome. As depicted in Fig. 5 A and B, protein levels of NLRP3, ASC, and cleaved caspase-1 were significantly upregulated 24 h after SAH, and the increment of these proteins was substantially reversed by treatment with OZ (*P*<0.001, *P*<0.001, and *P* = 0.0011). Furthermore, western blot results indicated that the SAH-induced enhancement of mature IL-1β and IL-18 were distinctly attenuated by OZ treatment (Fig. 5a, b, both *P* < 0.001).

Consistent with the observation in vivo, OxyHb incubation markedly upregulated the levels of NLRP3, ASC, cleaved caspase-1, mature IL-1β, and mature IL-18, which was significantly suppressed by pre-incubating the OxyHb-primed neurons with 600 nM of OZ (Fig. 5c, d, all *P* < 0.001).

### Inhibition of TAK1 blocks NF-κB activation after SAH

**Fig. 6:**
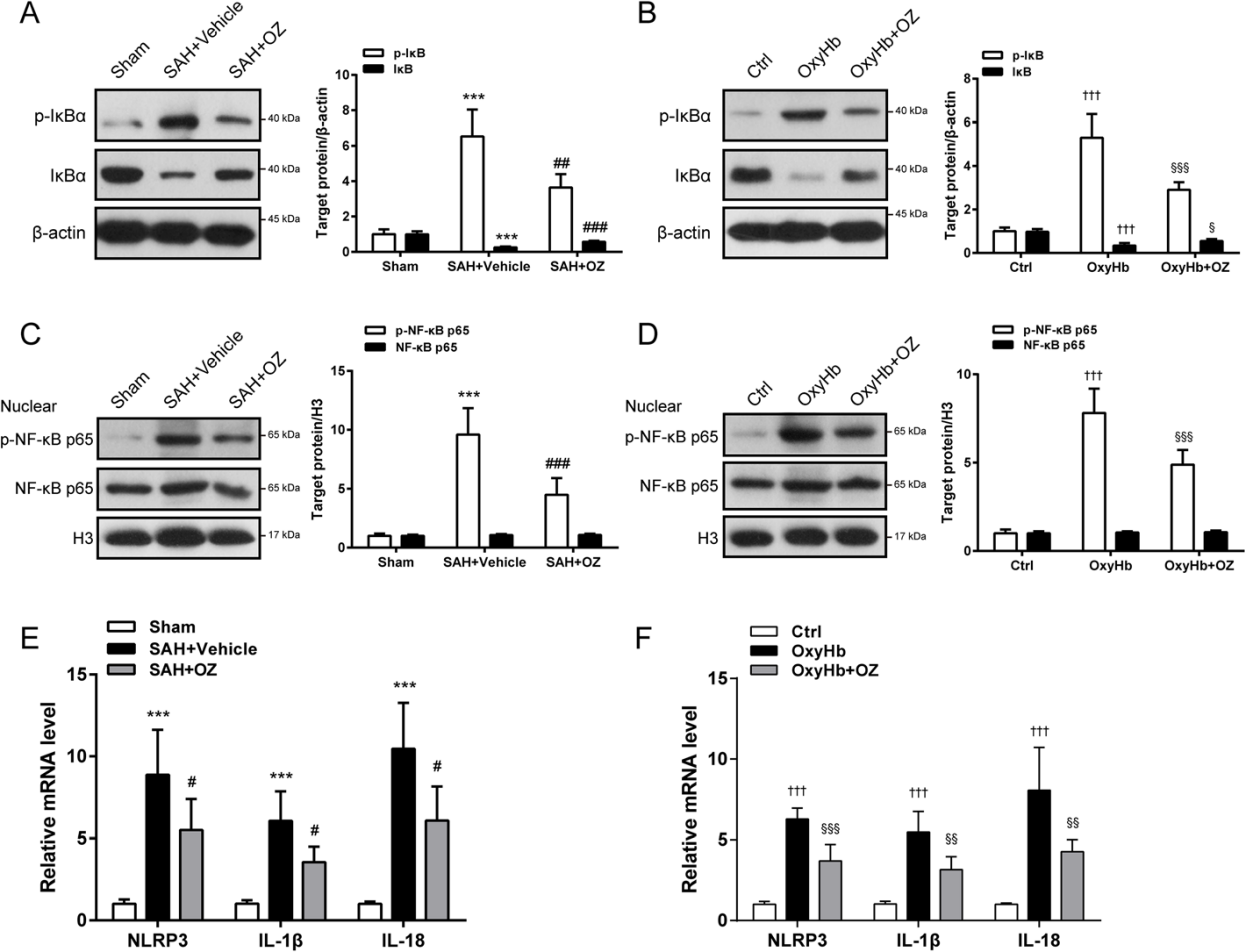
OZ treatment inhibits SAH-induced NF-κB p65 activation. **a**, **b** Western blot analysis of p-IκBa, IκBa in treated mice and primary neurons. OZ treatment significantly restrained the phosphorylation of IκBa and the degradation of IκBa induced by SAH. **c**, **d** Nuclear fractions of brain tissue and neurons were extracted. Protein levels of nuclear p-NF-κB p65 and NF-κB p65 in treated mice and primary neurons. OZ administration reduced SAH-induced enhancement of nuclear p-NF-κB p65. **e**, **f** mRNA levels of NLRP3, IL-β, and IL-18 in treated mice and primary neurons. Data are expressed as mean ± SD, *n* = 5 per group. ***P < 0.001 vs Sham group; ^#^*P* < 0.05, ^##^*P* < 0.01, ^###^*P* < 0.001 vs SAH+Vehicle group. ^†††^*P* < 0.001 vs Control group; ^§^*P* < 0.05, ^§§^*P* < 0.01, ^§§§^*P* < 0.001 vs OxyHb group

NF-κB signaling is required for the activation of NLRP3 inflammasome [13]. We then elucidate the effect of TAK1 inhibition on NF-κB signaling under SAH conditions. Our results revealed that enhanced phosphorylation of IκBа was detected in SAH mice and OxyHb-treated neurons, which was abolished by treatment with OZ (Fig. 6a, b, *P* = 0.0015 and *P* < 0.001). The basal level of IκBа was substantially reduced in SAH mice compared to that in sham mice, and reversed in OZ treatment groups (Fig. 6a, *P* < 0.001). A similar trend was detected in in vitro experiments (Fig. 6b, *P* = 0.0321). These results implied that OZ administration prevented the SAH-induced IκBа degradation. Moreover, there was a significant augmentation of nuclear phosphorylated NF-κB p65 (p-NF-κB p65) in SAH mice and OxyHb-treated neurons, and these increments were abrogated by application of OZ (Fig. 6c, d, both *P*<0.001). The expression of NF-κB target genes NLRP3, IL-1β, and IL-18 were also measured both in vivo and in vitro. We found that mRNA levels of NLRP3, IL-1β, and IL-18 were induced at 24 h after SAH, but these increments were impaired after treatment with OZ (Fig. 6e, *P* = 0.0426, *P* = 0.0151 and *P* = 0.0128). Similar to the observation in SAH mice, pre-treatment with OZ abolished the elevation of NLRP3, IL-1β, and IL-18 mRNA expressions in OxyHb-treated neurons (Fig. 6f, *P*<0.001, *P* = 0.0036 and *P* = 0.0071).

### Knockdown of endogenous TAK1 alleviated brain injury and inflammatory response after SAH

**Fig. 7:**
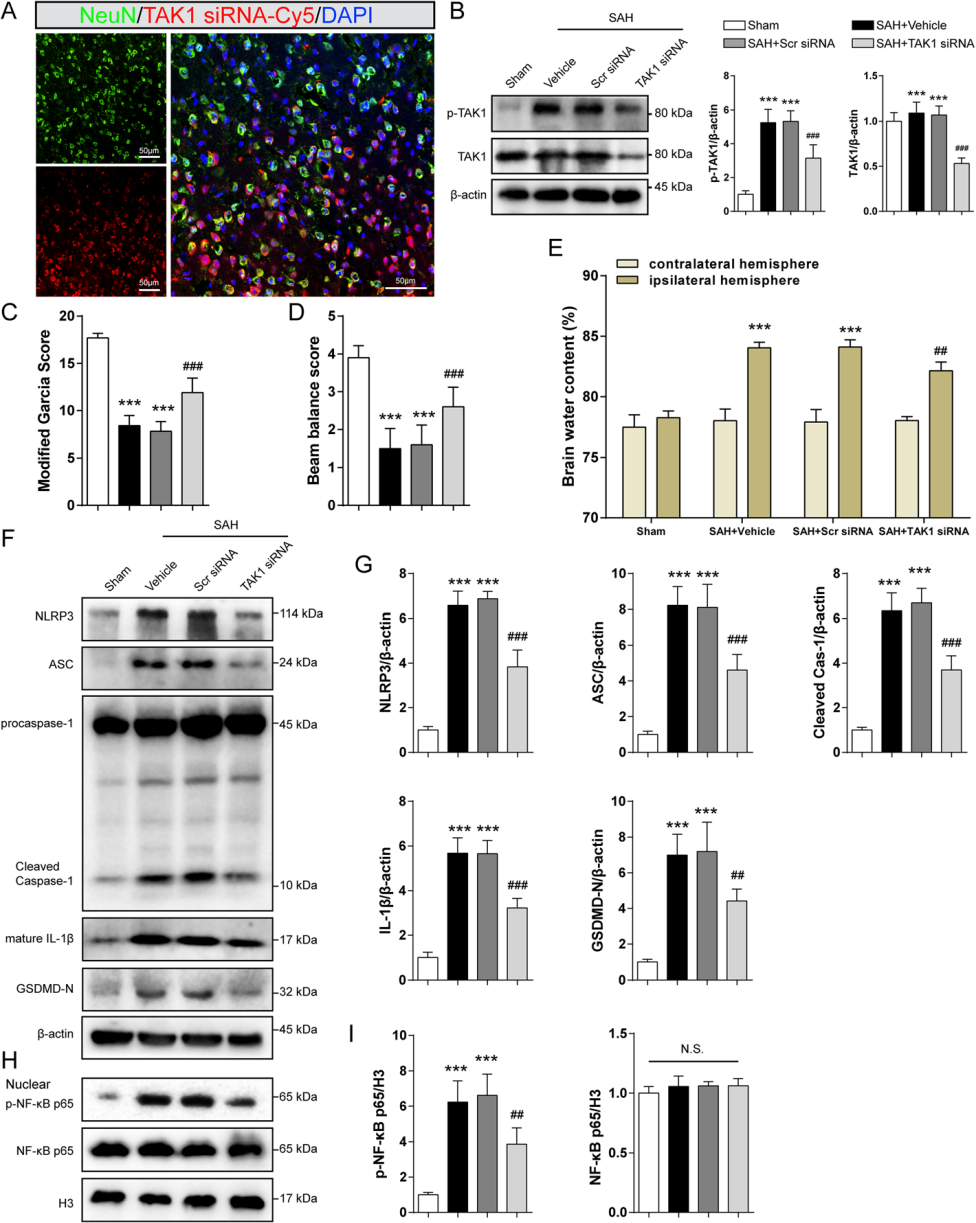
Effect of TAK1 knockdown on brain injury and inflammatory response after SAH. To knockdown of TAK1 in vivo, SAH mice were i.c.v injected with TAK1 siRNA. **a** Colocalization of Cy5-conjugated TAK1 siRNA with neurons (NeuN) at 24 h after SAH. **b** Western blotting and quantitative analysis for p-TAK1 and TAK1. **c** Modified Garcia score, **d** Beam balance test, and **e** brain water content in Sham, SAH+Vehicle, SAH+Scr siRNA, and SAH+TAK1 siRNA groups at 24 h after SAH. **f**–**i** Immunoblotting analysis and quantitation for NLRP3, ASC, cleaved caspase-1, IL-1β, GSDMD, nuclear p-NF-κB p65, and NF-κB p65 in treated mice. Data are expressed as mean ± SD, *n* = 5 in each group. ***P < 0.001 vs Sham group; ^##^*P* < 0.01, ^###^*P* < 0.001 vs SAH+Scr siRNA group. N.S., no significant difference. Scale bar: 50 μm

To further verify the role of TAK1 post-SAH, TAK1 siRNA was administrated i.c.v to silence endogenous TAK1. Double immunostaining showed that Cy5-conjugated TAK1 siRNA was co-stained with NeuN (Fig. 7a). TAK1 siRNA i.c.v injection significantly decreased the expression of p-TAK1 and TAK1 in the ipsilateral hemisphere of SAH mice (Fig. 7b, all *P*<0.001). These results suggest that TAK1 siRNA could transfect into neurons and knock down TAK1 expression. Genetic knockdown of TAK1 mitigated neurological deficits and brain edema at 24 h post-SAH (Fig. 7c–e, *P* < 0.001, *P* < 0.001 and *P=*0.0014). Moreover, treatment with TAK1 siRNA significantly restrained the expression of NLRP3, ASC, cleaved caspase-1, mature IL-1β, and GSDMD-N when compared to the SAH+Scr siRNA group (Fig. 7f, g, for GSDMD-N: *P=*0.004, for others: *P* < 0.001). TAK1 siRNA administration markedly reversed the enhancement of nuclear p-NF-κB p65 expression induced by SAH (Fig. 7h, i, *P*= 0.0019).

### OZ treatment inhibits NLRP3 inflammasome activation by reducing ROS production following SAH

**Fig. 8:**
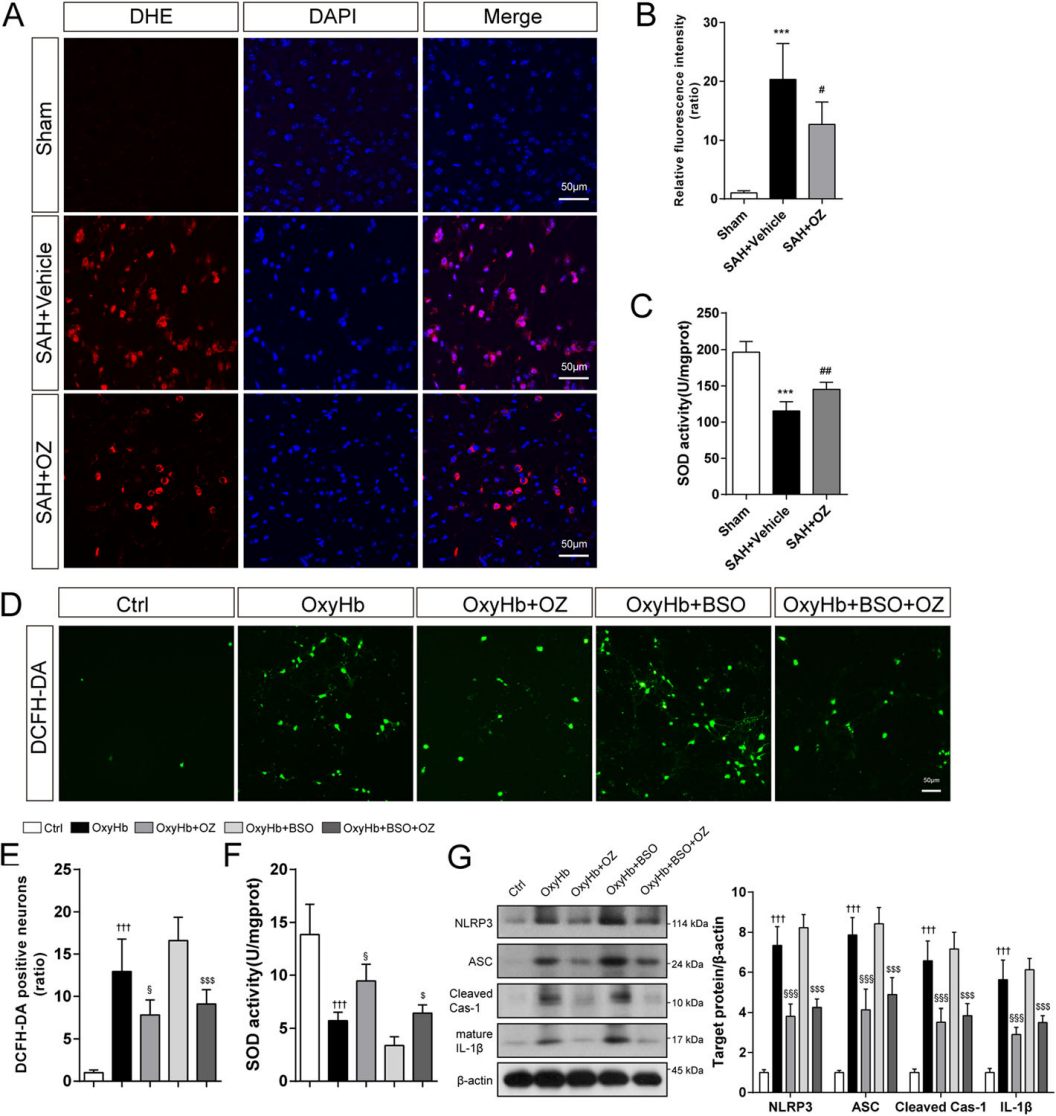
OZ treatment mitigates oxidative stress following SAH. **a** DHE staining in the ipsilateral cortex of mice brain and **b** quantitative analysis of DHE fluorescence intensity. **c** Assessment of SOD activity in brain tissues. **d** Treated neurons were incubated with 5 μM DCFH-DA to detect intracellular ROS levels and **e** quantitative analyses of the number of DCFH-DA-positive neurons. OZ administration notably reduced OxyHb- and OxyHb+BSO- induced increment of DCFH-DA-positive neurons. **f** Determination of SOD activity in neurons. **g** Western blot and quantitative analysis of NLRP3, ASC, cleaved caspase-1, IL-β in treated neurons. Data are expressed as mean ± SD, *n* = 5 per group. ***P < 0.001 vs Sham group; ^#^*P* < 0.05, ^##^*P* < 0.01 vs SAH+Vehicle group. ^†††^*P* < 0.001 vs Control group; ^§^*P* < 0.05, ^§§§^*P* < 0.001 vs OxyHb group; ^$^*P* < 0.05, ^$$$^*P* < 0.001 vs OxyHb+BSO group. Scale bar: 50 μm

Considering that NLRP3 inflammasome activation is closely related to ROS, we next detected that whether ROS was responsible for OZ-mediated NLRP3 inflammation. Compared with the sham group, the fluorescence density of DHE was 20.3-fold higher in the vehicle-treated group 24h after SAH, whereas OZ injection led to a 37.3% reduction in DHE fluorescence density (Fig. 8a, b, *P* = 0.0345). Additionally, OZ treatment notably reversed the increment of total SOD level induced by SAH attack (Fig. 8c, *P* =0.0072). The level of intracellular reactive oxygen species in primary neurons was assessed by DCFH-DA staining. As illustrated in Fig. 8d, e, DCFH-DA positive neurons were upregulated by 12.9-fold in the OxyHb-treated group, and OZ pre-treatment notably reduced the DCFH-DA positive neurons by 39.5% (*P* = 0.0208). l-Buthionine-sulfoximine (BSO) is an inhibitor of glutathione synthetase, which causes ROS accumulation in the cytoplasm. OZ administration significantly decreased the number of DCFH-DA positive neurons in the OxyHb+BSO+OZ group compared with those in the OxyHb+BSO group (Fig. 8d, e, *P* < 0.001). Consistently, we found that OZ could reverse BSO-induced down-regulation of SOD in OxyHb treated neurons (Fig. 8f, *P* = 0.0431). Western blot results showed that the effect of BSO on the expressions of NLRP3, ASC, cleaved caspase-1, and mature IL-1β was also countered by OZ (Fig. 8g, all *P* < 0.001). These findings indicate that TAK1 inhibition can mitigate NLRP3 inflammasome activation via attenuating ROS production.

### Blockade of TAK1 inhibits lysosomal rupture in oxyHb-treated neurons

**Fig. 9:**
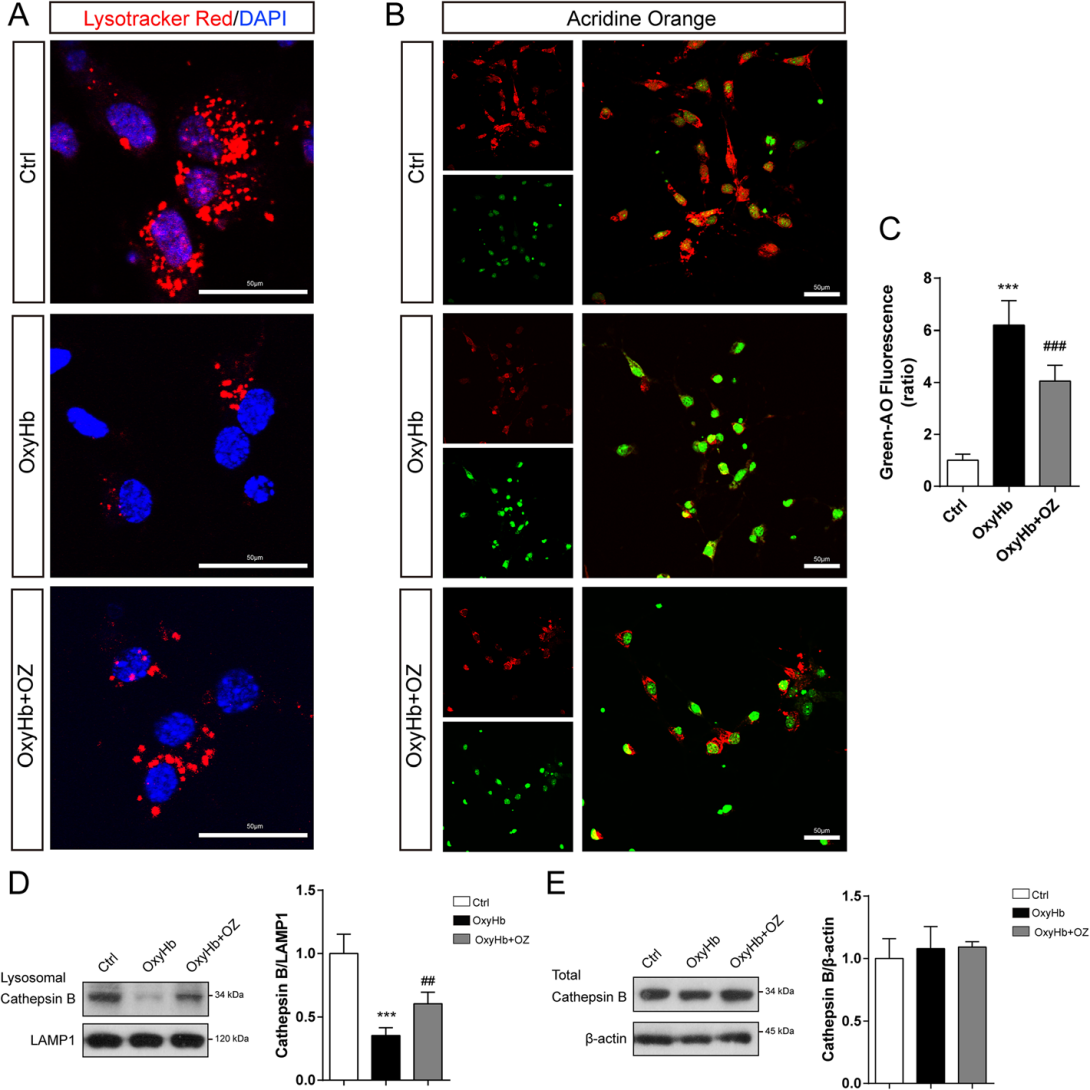
OZ treatment inhibits lysosomal rupture in OxyHb-treated neurons. **a** Ctrl-, OxyHb-, and OxyHb+OZ-treated neurons were incubated with 100 nM Lyso-Tracker Red for 30 min to assess lysosomal stability. **b** Neurons of the three groups were incubated with 2 μg/ml Acridine Orange for 30 min to evaluate lysosomal dysfunction. **c** Quantitative analyses of the green fluorescence intensity of Acridine Orange with a microplate reader. **d**, **e** Western blot and quantitative analysis of lysosomal and total cathepsin B in the three groups. Data are expressed as mean ± SD, *n* = 5 per group. ***P < 0.001 vs Control group; ^##^*P* < 0.01, ^###^*P* < 0.001 vs OxyHb group. Scale bar: 50 μm

Lysosomal dysfunction could induce NLRP3 inflammasome activation [31]. We next tested whether inhibition of TAK1 attenuates lysosome rupture in OxyHb-injured neurons. We used Lyso-Tracker Red staining to assess lysosomal stability. As shown in Fig. 9a, OxyHb-treated neurons displayed a loss of Lyso-Tracker fluorescent signal compared to the control group, while OZ administration reversed this trend. Acridine Orange yielded red fluorescence when accumulated within the lysosome and green fluorescence when released from ruptured lysosome and diffused into the cytosol and nuclei [30]. We performed Acridine Orange staining to detect lysosomal destabilization. OxyHb incubation enhanced green fluorescence in neurons, which was inhibited by OZ treatment (Fig. 9 b and c, *P* < 0.001). Western blot results showed that lysosomal cathepsin B was reduced in OxyHb-treated neurons, while OZ treatment significantly upregulated lysosomal cathepsin B content (Fig. 9d, *P* = 0.0081). The total amount of cathepsin B had no significant changes among the groups (Fig. 9e). These results suggest that blockade of TAK1 may restrain lysosomal rupture and reduce cathepsin B releasing into the cytosol.

## Discussion

**Fig. 10:**
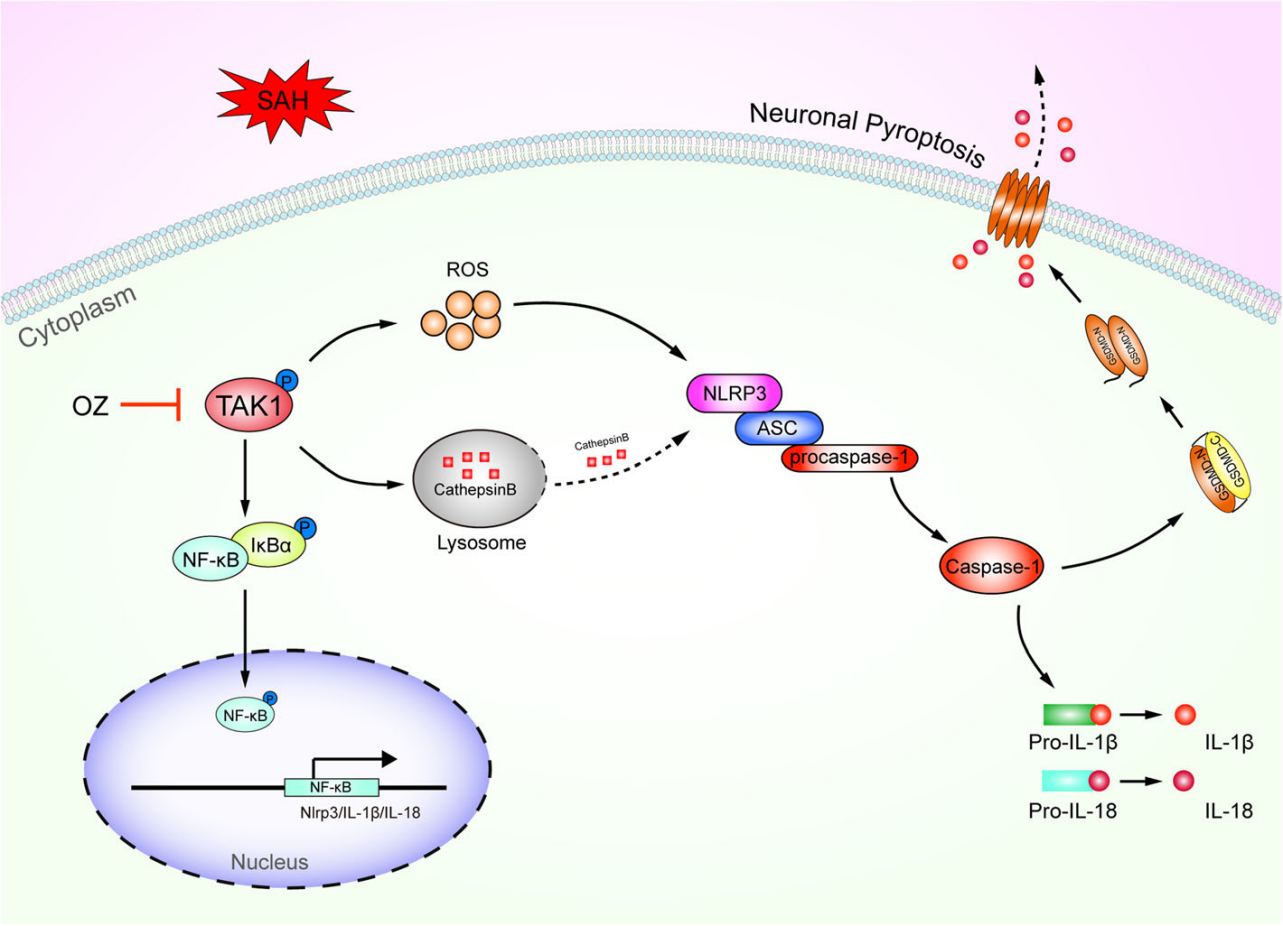
Schematic diagram for TAK1-mediated neuronal pyroptosis in EBI following SAH. The p-TAK1 level is upregulated following SAH ictus. Inhibition of TAK1 by OZ treatment reduces ROS production and prevents lysosomal cathepsin B releasing into the cytoplasm. ROS and cathepsin B could activate NLRP3 inflammasome. Blockade of TAK1 mitigates neuronal pyroptosis via inhibition of NLRP3 inflammasome and NF-κB signaling pathway. Treatment with OZ shows remarkable therapeutic effects on EBI following SAH

The present study demonstrated that p-TAK1 (Thr184/187) was triggered in neurons after experimental SAH in mice. Pharmacological blockade of TAK1 with OZ attenuated neurological impairments, brain edema, and BBB disruption. Administration of OZ remarkably reduced neuronal pyroptosis in EBI through inhibiting NLRP3 inflammasome activation. The mechanism of OZ in inhibition of NLRP3 inflammasome activation may be related to attenuating ROS production and protecting lysosomal integrity (Fig. 10).

In SAH rats, Zhang and colleagues demonstrated that p-TAK1 (Thr187) rather than p-TAK1 (ser439) was upregulated in neurons [7]. In a neonatal model of hypoxic-ischemic injury, the p-TAK1 level was increased in the brain 3 h after injury [32]. Consistent with these studies, we found that p-TAK1 (Thr184/187) was activated in neurons following SAH using the mouse SAH model. Yet, a study examining the functional role of TAK1 in ischemic stroke mice showed that the level of p-TAK1 (Thr187) was downregulated 4 h after ischemia [33]. The differences in levels of TAK1 expression between these studies might be due to model differences, animal species, and measurement time points.

There is a tremendous interest in TAK1 inhibition as a therapeutic application for inflammatory-related disorders, such as progressive kidney disease, ischemic stroke, and SAH [7, 27, 33, 34]. In our experiments, TAK1 inhibition was accomplished by treatment with OZ, a highly selective inhibitor of TAK1. OZ selectively inhibits the catalytic activity of TAK1 but not other MAP3Ks [35]. OZ lost its efficacy in TAK1^−/−^ neurons further confirmed its specificity. A previous study showed that inhibition of TAK1 could ameliorate neurological deficits, but failed to attenuate SAH-induced brain edema [7]. However, in our study, inhibition of TAK1 could reverse both neurological dysfunction and cerebral edema. Notwithstanding the differences in attenuating brain edema, both researches suggest that TAK1 might be a promising molecular target for the treatment of SAH.

After SAH, hemoglobin could metabolize to oxyhemoglobin, heme, and globin. Oxyhemoglobin and heme are toxic to neurons. In this study, we used oxyhemoglobin to mimic in vitro SAH model and found that oxyhemoglobin could induce p-TAK1 expression. Previous reviews indicated that TAK1 could be activated by heme via the TLR4/MYD88 pathway [36, 37]. As a key signaling molecule in innate immune signaling pathways, TAK1 could also be activated by other DAMPs, including thrombin, fibrinogen, IL-1β, TNF-α, and damaged DNA [6, 38, 39]. The activated TAK1 triggers phosphorylation and activation of MAPKs (p38, JNK, and ERK) and IkB kinase (IKK)–NF-kB pathways [6, 38, 39]. Indeed, TAK1 activation was found to promote ischemic brain injury by activating JNK and/or p38 and NF-kB[40]. We here observed that OZ treatment prevented the degradation of IkB and phosphorylation of p65 NF-kB in SAH mice and OxyHb-treated neurons, suggesting that TAK1 activates NF-kB signaling following SAH.

Pyroptosis, a gasdermin-mediated programmed necrosis, is well studied in various diseases in recent years [14, 41, 42]. After cleavage by inflammatory caspases, gasdermin was broken into two domains, the N-terminal gasdermin (GSDMD-N) and C-terminal gasdermin (GSDMD-C) [14, 43]. GSDMD-N is the pro-pyroptotic fragment of GSDMD, which can bind to the phosphoinositides in the plasma membrane and oligomerizes into membrane pores of about 12–14 nm [14, 43]. Pore formation results in rapid loss of membrane integrity, extracellular spilling of intracellular contents, and ultimately cell death [14, 43]. Recent studies discovered that TAK1 inactivation in macrophages triggers a caspase-8-mediated inflammatory pathway involving GSDMD [15, 44]. Currently, knowledge regarding neuronal pyroptosis in EBI after SAH has been limited. To address this gap, we examined whether TAK1 could influence neuronal pyroptosis following SAH in this study. We here showed the first evidence that SAH insults increased the GSDMD-positive neurons, GSDMD pore in the neuronal membrane, and protein expression of GSDMD-N in mouse brains, whereas 3 μg of OZ suppressed those effects. It is also worth mentioning that the secretion of IL-1β and IL-18 was alleviated by OZ administration. These results suggest that TAK1 inhibition could reduce neuronal pyroptosis after SAH. A recent study reported that IL-18, which is induced by microglial TAK1 activation in the obese mouse brainstem, could act with the IL-18 receptor on endothelial cells to decrease the activity of eNOS, thereby resulting in endothelial dysfunction [45]. So, in our study, IL-18 secretion induced by neuronal pyroptosis might aggravate BBB permeability. It is known that IL-1β could activate TAK1 [46]. In this study, we demonstrated that rIL-1β could upregulate the protein levels of p-TAK1 and components of NLRP3 inflammasome in primary neurons. Accordingly, neuronal pyroptosis might amplify extracellular immune responses by promoting secretion of inflammatory cytokines through membrane pores.

NLRP3 inflammasome, which consists of NLRP3, adaptor apoptosis-associated speck-like protein (ASC), and pro-caspase-1, is the best characterized inflammasome [13, 47]. It is well-established that NLRP3 activation requires two signals: first, increased transcription of the NLRP3, IL-1β, and IL-18 genes, which dependent on the activation of NF-kB through pattern recognition receptors, and second, NLRP3-activating stimuli, such as extracellular ATP, K^+^ efflux, endogenous danger-associated molecules [13]. Our previous work and others have demonstrated that NLRP3 inflammasome activation could mediate cell pyroptosis [48, 49]. Evidence has shown that TAK1 regulates lysosome rupture-induced and extracellular osmolarity decrease-induced NLRP3 inflammasome activation [9, 11, 12]. We speculate that TAK1 may trigger neuronal pyroptosis through activating NLRP3 inflammasome. Indeed, our real-time PCR results showed that gene expression of NLRP3, IL-1β, and IL-18 in neurons was suppressed by OZ administration, and the protein expression of NLRP3, ASC, and cleaved caspase-1 was also reduced, indicating that TAK1 inhibition prevents neuronal NLRP3 activation. Our results were consistent with a previous report indicating that OZ treatment inhibits the NLRP3 inflammasome pathway by targeting TAK1 in the cartilaginous endplate degeneration model [50]. Nevertheless, our data conflict with a report that TAK1 restricts spontaneous NLRP3 activation in myeloid cells [51]. We found that OZ treatment protects the lysosomal membrane and reducing cathepsin B releasing into the cytosol. However, our findings are paradoxical with a report by Sakamachi et al. [52], in which they found that TAK protects macrophage lysosomal integrity. Of interest, we detected a significant reduction of ROS levels in neurons after treatment with OZ. ROS and cathepsin B are the endogenous danger-associated molecules, which have been shown to induce NLRP3 inflammasome activation [53, 54]. In view of this, TAK1 might activate NLRP3 inflammasome indirectly after SAH. Additional works are needed to elucidate the specific ways of TAK1 activating NLRP3 inflammasome.

In summary, TAK1 inhibition could provide a neuroprotective effect against EBI following SAH. The mechanisms involve the prevention of neuronal NLRP3 inflammasome activation and pyroptosis. These findings indicate that TAK1 may be a therapeutic target for SAH.

## Supplementary Information

**Additional file 1: Fig. S1**. Experimental design and animal groups. DHE, dihydroethidium; IF, immunofluorescence; i.c.v, intracerebroventricular; MWM, Morris water maze; OZ, 5Z-7-oxozeaenol; RT-PCR, real-time polymerase chain reaction; SAH, subarachnoid hemorrhage; siRNA, short interfering RNA; Scr siRNA, scrambled siRNA.TEM, transmission electron microscope; WB, western blot.

**Additional file 2: Fig. S2**. Schematic diagram of regions of interest (ROIs) in ipsilateral cortex. Four small black square within coronal section of ipsilateral brain indicated the location of where the immunofluorescence staining images were taken.

**Additional file 3: Fig. S3**. Mortality and SAH grade. (A) Animal usage and mortality of all the experimental groups. (B) Representative brain images of sham-operated and SAH mice. (C) SAH grade scores of all SAH groups. No significant difference in SAH severity was found among groups. Data are expressed as mean ± SD. N.S., no significant difference; OZ, 5Z-7-oxozeaenol; Scr siRNA, scrambled siRNA.

**Additional file 4: Fig. S4**. OZ treatment inhibited p-TAK1 and TAK1 expression following SAH. (A) Western blot analysis of p-TAK1 and TAK1 in ipsilateral cortex from Sham, SAH+Vehicle, and SAH+OZ (1μg and 3μg) groups. (B) Quantification analysis of the proteins. Data are expressed as mean ± SD, n = 5 in each group. ***P < 0.001 vs Sham group; ^##^*P* < 0.01, ^###^*P* < 0.001 vs SAH+Vehicle group.

**Additional file 5: Fig. S5**. OZ pre-treatment inhibited p-TAK1 and TAK1 expression in vitro. Primary neurons were incubated with OxyHb (25 μM) to mimic SAH condition in vitro. To inhibit TAK1, neurons were pre-treated with OZ (600 nM) for 2h before OxyHb incubation. (A) Immunoblots and (B) densitometry analysis of p-TAK1 and TAK1 in Control, OxyHb and OxyHb+OZ groups. Data are expressed as mean ± SD, n = 5 in each group. ***P < 0.001 vs Control group; ^##^*P* < 0.01, ^###^*P* < 0.001 vs OxyHb group.

**Additional file 6: Fig. S6**. Effect of rIL-1β on p-TAK1 and NLRP3 inflammasome in vitro. Primary neurons were treated with 10 ng/ml rIL-1β for 24h. (A) Immunoblots and (B) quantitative analysis of p-TAK1, NLRP3, ASC and Cleaved Caspase-1 in Control and rIL-1β-treated neurons. Data are expressed as mean ± SD, n = 5 in each group. **P < 0.01, ***P < 0.001 vs Control group.

**Additional file 7: Table S1**. Physiological variables in the experimental mice.

## Data Availability

All data generated or analyzed during this study are included in this published article and its supplementary files.

## Abbreviations

ASC: poptosis-associated speck-like protein containing a caspase recruitment domain
BBB: Brain-blood barrier
BSO: l-Buthionine-sulfoximine
Caspase-1: Cysteinyl aspartate-specific proteinase-1
DCFH-DA: 2′,7′-Dichlorofluorescin diacetate
DHE: Dihydroethidium
EBI: Early brain injury
ELISA: Enzyme-linked immuno sorbent assay
GSDMD: Gasdermin D
IL-1β: Interleukin-1β
IL-18: Interleukin-18
ICA: Internal carotid artery
LAMP1: Lysosomal-associated membrane protein 1
MAPKs: Mitogen-activated protein kinases
MAP3K: Mitogen-activated protein kinase kinase kinase
NF-κB: Nuclear factor-κB
NLRP3: Nucleotide-binding oligomerization domain (NOD)-like receptor pyrin domain containing 3
OZ: 5Z-7-oxozeaenol
OxyHb: Oxyhemoglobin
ROS: Reactive oxygen species
rIL-1β: IL-1β recombinant protein
SAH: Subarachnoid hemorrhage
siRNA: Short interfering RNA
TAK1: Transforming growth factor-β-activated kinase 1
