# Supplementary material for: TAK1 mediates neuronal pyroptosis in early brain injury after subarachnoid hemorrhage
Source: J Neuroinflammation. 2021 Aug 30;18:188. doi: 10.1186/s12974-021-02226-8 (PMC8406585; doi:10.1186/s12974-021-02226-8)
Supplement: Supplementary file 1 — Additional file 1: Fig. S1. Experimental design and animal groups. DHE, dihydroethidium; IF, immunofluorescence; i.c.v, intracerebroventricular; MWM, Morris water maze; OZ, 5Z-7-oxozeaenol; RT-PCR, real-time polymerase chain reaction; SAH, subarachnoid hemorrhage; siRNA, short interfering RNA; Scr siRNA, scrambled siRNA.TEM, transmission electron microscope; WB, western blot. [file 12974_2021_2226_MOESM1_ESM.docx]

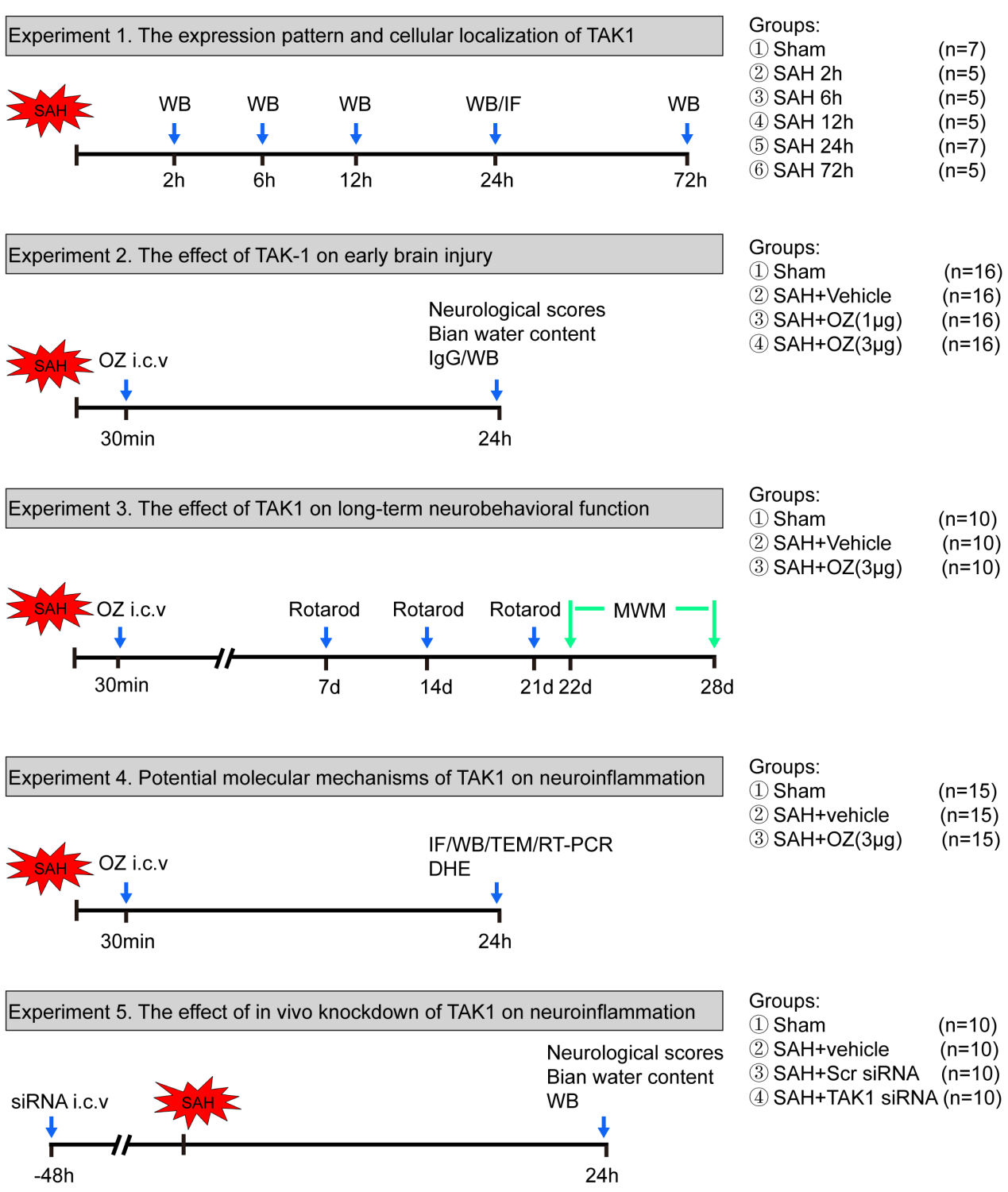


**Fig.S1 Experimental design and animal groups.** DHE, dihydroethidium; IF, immunofluorescence; i.c.v, intracerebroventricular injection; MWM, Morris water maze; OZ, 5Z-7-oxozeaenol; RT-PCR, real-time polymerase chain reaction; SAH, subarachnoid hemorrhage; siRNA, short interfering RNA; Scr siRNA, scrambled siRNA.TEM, transmission electron microscope; WB, western blot.
