# Supplementary material for: TAK1 mediates neuronal pyroptosis in early brain injury after subarachnoid hemorrhage
Source: J Neuroinflammation. 2021 Aug 30;18:188. doi: 10.1186/s12974-021-02226-8 (PMC8406585; doi:10.1186/s12974-021-02226-8)
Supplement: Supplementary file 2 — Additional file 2: Fig. S2. Schematic diagram of regions of interest (ROIs) in ipsilateral cortex. Four small black square within coronal section of ipsilateral brain indicated the location of where the immunofluorescence staining images were taken. [file 12974_2021_2226_MOESM2_ESM.docx]

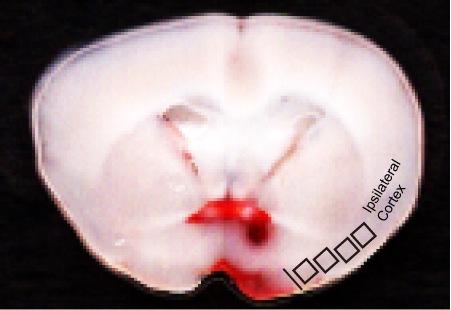


**Fig.S2 Schematic diagram of regions of interest (ROIs) in ipsilateral cortex.** Four small black square within coronal section of ipsilateral brain indicated the location of where the immunofluorescence staining images were taken.
