# Supplementary material for: TAK1 mediates neuronal pyroptosis in early brain injury after subarachnoid hemorrhage
Source: J Neuroinflammation. 2021 Aug 30;18:188. doi: 10.1186/s12974-021-02226-8 (PMC8406585; doi:10.1186/s12974-021-02226-8)
Supplement: Supplementary file 3 — Additional file 3: Fig. S3. Mortality and SAH grade. (A) Animal usage and mortality of all the experimental groups. (B) Representative brain images of sham-operated and SAH mice. (C) SAH grade scores of all SAH groups. No significant difference in SAH severity was found among groups. Data are expressed as mean ± SD. N.S., no significant difference; OZ, 5Z-7-oxozeaenol; Scr siRNA, scrambled siRNA. [file 12974_2021_2226_MOESM3_ESM.docx]

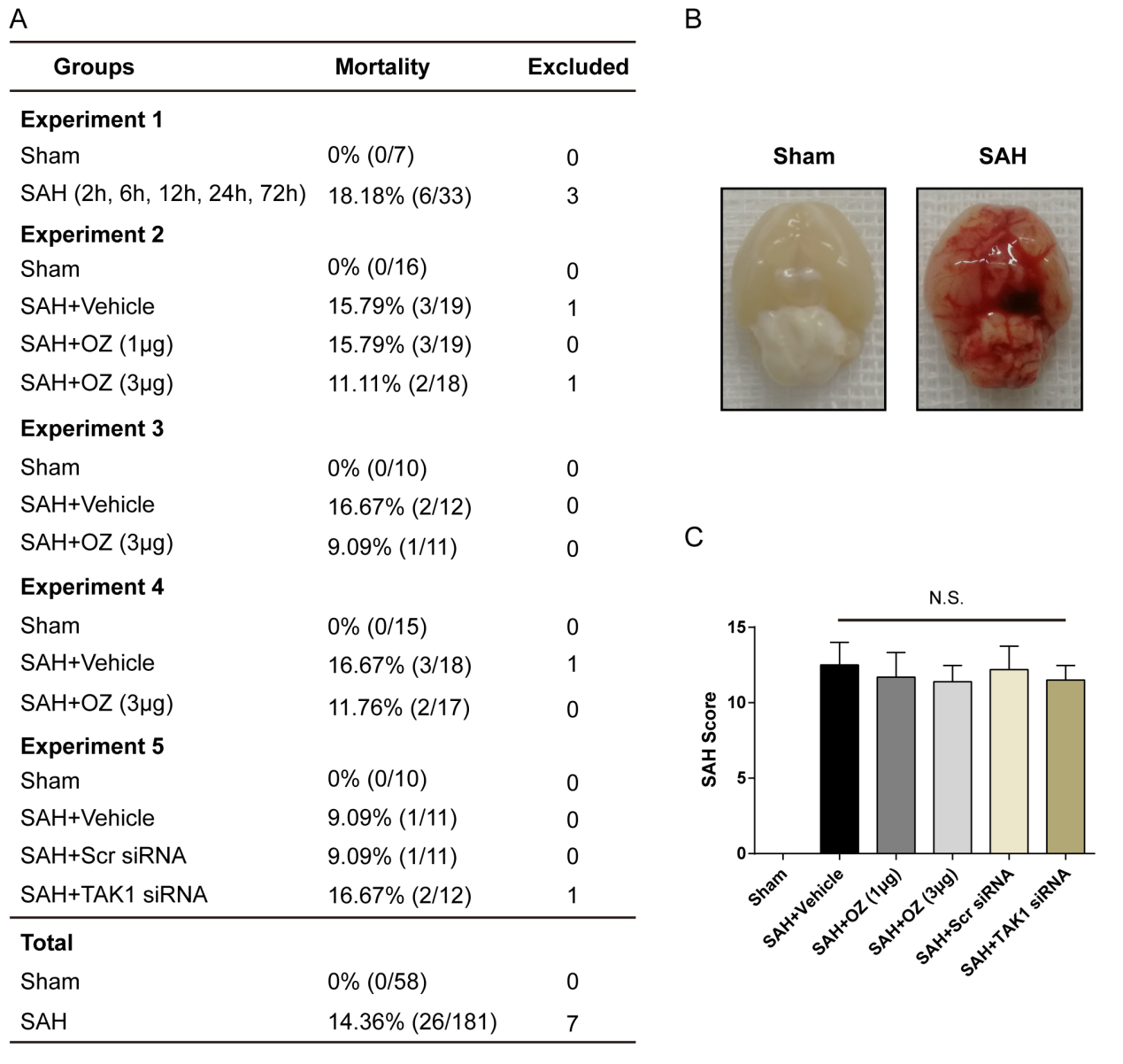


**Fig. S3 Mortality and SAH grade.** (A) Animal usage and mortality of all the experimental groups. (B) Representative brain images of sham-operated and SAH mice. (C) SAH grade scores of all SAH groups. No significant difference in SAH severity was found among groups. Data are expressed as mean ± SD. N.S., no significant difference; OZ, 5Z-7-oxozeaenol; Scr siRNA, scrambled siRNA.
