# Supplementary material for: TAK1 mediates neuronal pyroptosis in early brain injury after subarachnoid hemorrhage
Source: J Neuroinflammation. 2021 Aug 30;18:188. doi: 10.1186/s12974-021-02226-8 (PMC8406585; doi:10.1186/s12974-021-02226-8)
Supplement: Supplementary file 4 — Additional file 4: Fig. S4. OZ treatment inhibited p-TAK1 and TAK1 expression following SAH. (A) Western blot analysis of p-TAK1 and TAK1 in ipsilateral cortex from Sham, SAH+Vehicle, and SAH+OZ (1μg and 3μg) groups. (B) Quantification analysis of the proteins. Data are expressed as mean ± SD, n = 5 in each group. ***P < 0.001 vs Sham group; ##P < 0.01, ###P < 0.001 vs SAH+Vehicle group. [file 12974_2021_2226_MOESM4_ESM.docx]

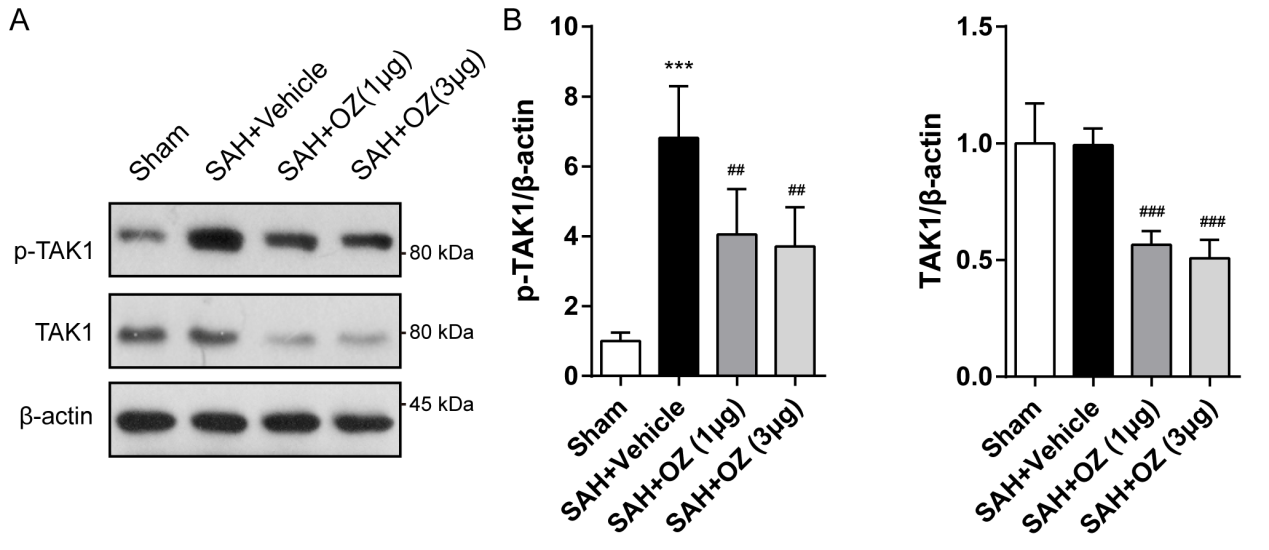


**Fig. S4 OZ treatment inhibited p-TAK1 and TAK1 expression following SAH.**

(A) Western blots analysis of p-TAK1 and TAK1 in ipsilateral cortex from Sham, SAH+Vehicle, and SAH+OZ (1μg and 3μg) groups. (B) Quantification analysis of the proteins. Data are expressed as mean ± SD, n = 5 in each group. ****P* < 0.001 vs Sham group; ^##^*P* < 0.01, ^###^*P* < 0.001 vs SAH+Vehicle group.
