# Supplementary material for: TAK1 mediates neuronal pyroptosis in early brain injury after subarachnoid hemorrhage
Source: J Neuroinflammation. 2021 Aug 30;18:188. doi: 10.1186/s12974-021-02226-8 (PMC8406585; doi:10.1186/s12974-021-02226-8)
Supplement: Supplementary file 5 — Additional file 5: Fig. S5. OZ pre-treatment inhibited p-TAK1 and TAK1 expression in vitro. Primary neurons were incubated with OxyHb (25 μM) to mimic SAH condition in vitro. To inhibit TAK1, neurons were pre-treated with OZ (600 nM) for 2h before OxyHb incubation. (A) Immunoblots and (B) densitometry analysis of p-TAK1 and TAK1 in Control, OxyHb and OxyHb+OZ groups. Data are expressed as mean ± SD, n = 5 in each group. ***P < 0.001 vs Control group; ##P < 0.01, ###P < 0.001 vs OxyHb group. [file 12974_2021_2226_MOESM5_ESM.docx]

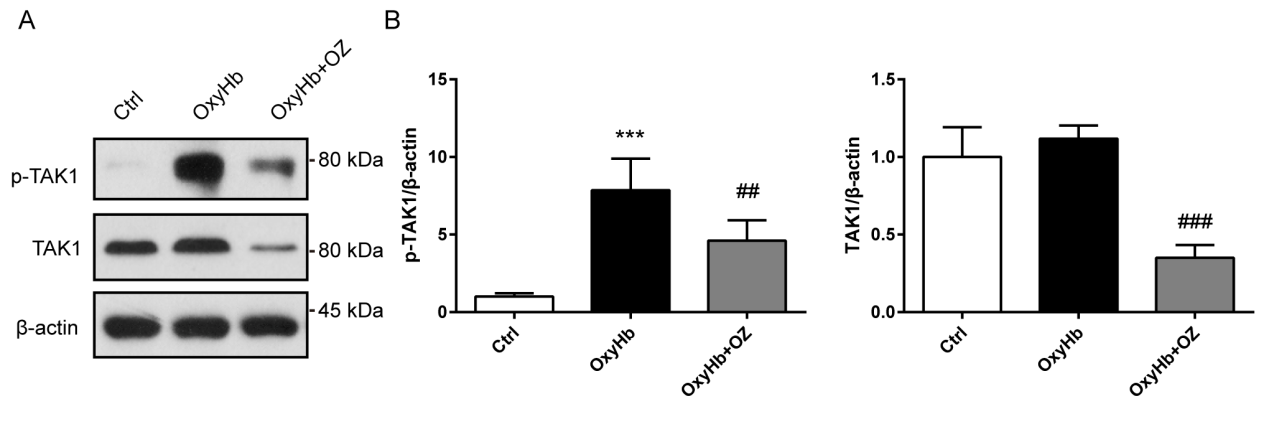


**Fig. S5 OZ pre-treatment inhibited p-TAK1 and TAK1 expression in vitro.**

Primary neurons were incubated OxyHb (25μM) to mimic SAH condition in vitro. To inhibit TAK1, neurons were pre-treated with OZ (600 nM) for 2h before OxyHb incubation. (A) Immunoblots and (B) densitometry analysis of p-TAK1 and TAK1 in control, OxyHb and OxyHb+OZ groups. Data are expressed as mean ± SD, n = 5 in each group. ****P* < 0.001 vs Control group; ^##^*P* < 0.01, ^###^*P* < 0.001 vs OxyHb group.
