# Supplementary material for: TAK1 mediates neuronal pyroptosis in early brain injury after subarachnoid hemorrhage
Source: J Neuroinflammation. 2021 Aug 30;18:188. doi: 10.1186/s12974-021-02226-8 (PMC8406585; doi:10.1186/s12974-021-02226-8)
Supplement: Supplementary file 6 — Additional file 6: Fig. S6. Effect of rIL-1β on p-TAK1 and NLRP3 inflammasome in vitro. Primary neurons were treated with 10 ng/ml rIL-1β for 24h. (A) Immunoblots and (B) quantitative analysis of p-TAK1, NLRP3, ASC and Cleaved Caspase-1 in Control and rIL-1β-treated neurons. Data are expressed as mean ± SD, n = 5 in each group. **P < 0.01, ***P < 0.001 vs Control group. [file 12974_2021_2226_MOESM6_ESM.docx]

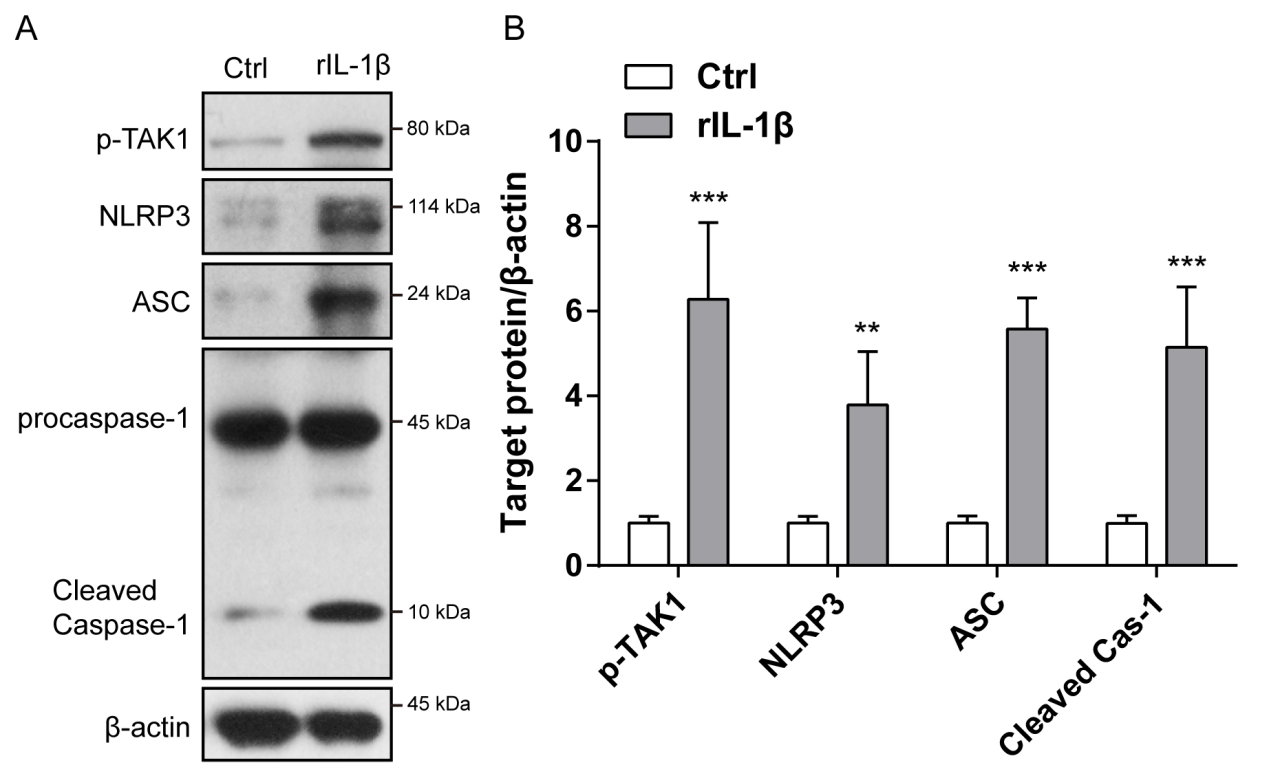


**Fig. S6 Effect of rIL-1β on p-TAK1 and NLRP3 inflammasome in vitro.**

Primary neurons were treated with 10 ng/ml rIL-1β for 24h. (A) Immunoblots and (B) quantitative analysis of p-TAK1, NLRP3, ASC and Cleaved Caspase-1 in Control and rIL-1β-treated neurons. Data are expressed as mean ± SD, n = 5 in each group. ****P* < 0.001 vs Control group.
