# Supplementary material for: TAK1 mediates neuronal pyroptosis in early brain injury after subarachnoid hemorrhage
Source: J Neuroinflammation. 2021 Aug 30;18:188. doi: 10.1186/s12974-021-02226-8 (PMC8406585; doi:10.1186/s12974-021-02226-8)
Supplement: Supplementary file 7 — Additional file 7: Table S1. Physiological variables in the experimental mice. [file 12974_2021_2226_MOESM7_ESM.docx]

**Table S1 Physiological variables in the experimental mice.**

|  | Sham | SAH+Vehicle | SAH+OZ | SAH+Scr siRNA | SAH+ TAK1 siRNA |
| --- | --- | --- | --- | --- | --- |
| PH | 7.33±0.04 | 7.32±0.03 | 7.32±0.04 | 7.35±0.06 | 7.37±0.09 |
| PO2,mmHg | 105.8±2.8 | 104.7±3.3 | 105.0±3.9 | 104.3±5.9 | 106.5±6.6 |
| PCO2,mmHg | 42.9±1.1 | 43.7±2.0 | 43.2±1.1 | 44.1±0.9 | 43.6±1.5 |
| MABP,mmHg | 105.8±4.4 | 104.5±2.7 | 104.3±4.5 | 106.2±4.9 | 107.3±7.5 |
| Plasma glucose, mg/dl | 119.2±4.2 | 118.7±4.9 | 122.5±5.9 | 124.0±7.0 | 125.2±5.9 |

There were no significant differences among groups in any of the parameters. SAH, subarachnoid hemorrhage; OZ, 5Z-7-oxozeaenol; Scr siRNA, scrambled siRNA; MABP, mean blood pressure.
